# Virtual reality—enhanced walking in people post-stroke: effect of optic flow speed and level of immersion on the gait biomechanics

**DOI:** 10.1186/s12984-023-01254-0

**Published:** 2023-09-25

**Authors:** Emma De Keersmaecker, Anke Van Bladel, Silvia Zaccardi, Nina Lefeber, Carlos Rodriguez-Guerrero, Eric Kerckhofs, Bart Jansen, Eva Swinnen

**Affiliations:** 1 Rehabilitation Research, Department of Physiotherapy, Human Physiology and Anatomy, Vrije Universiteit Brussel, Brussels, Belgium; 2 Center for Neurosciences (C4N), Vrije Universiteit Brussel, Brussels, Belgium; 3 Brussels Human Robotic Research Center (BruBotics), Vrije Universiteit Brussel, Brussels, Belgium; 4 Alliance research group REBI (Rehabilitation technology for people with a brain injury), Vrije Universiteit Brussel & Ghent University, Brussels, Belgium; 5 Faculty of Medicine and Health Sciences, Department Rehabilitation Sciences, Campus UZ Gent, Ghent, Belgium; 6 Department of Electronics and Informatics, Engineering Sciences, Vrije Universiteit Brussel, Brussels, Belgium; 7 Movement and Nutrition for Health and Performance, Vrije Universiteit Brussel, Brussels, Belgium; 8 Department of Mechanical Engineering, KU Leuven, Heverlee, Leuven, Belgium; 9 Imec, Leuven, Belgium

**Keywords:** Virtual reality, Optic flow, Gait biomechanics, Stroke

## Abstract

**Background:**

Optic flow—the apparent visual motion experienced while moving—is absent during treadmill walking. With virtual reality (VR), optic flow can be controlled to mediate alterations in human walking. The aim of this study was to investigate (1) the effects of fully immersive VR and optic flow speed manipulation on gait biomechanics, simulator sickness, and enjoyment in people post-stroke and healthy people, and (2) the effects of the level of immersion on optic flow speed and sense of presence.

**Methods:**

Sixteen people post-stroke and 16 healthy controls performed two VR-enhanced treadmill walking sessions: the semi-immersive GRAIL session and fully immersive head-mounted display (HMD) session. Both consisted of five walking trials. After two habituation trials (without and with VR), participants walked three more trials under the following conditions: matched, slow, and fast optic flow. Primary outcome measures were spatiotemporal parameters and lower limb kinematics. Secondary outcomes (simulator sickness, enjoyment, and sense of presence) were assessed with the Simulator Sickness Questionnaire, Visual Analogue Scales, and Igroup Presence Questionnaire.

**Results:**

When walking with the immersive HMD, the stroke group walked with a significantly slower cadence (-3.69strides/min, p = 0.006), longer stride time (+ 0.10 s, p = 0.017) and stance time for the unaffected leg (+ 1.47%, p = 0.001) and reduced swing time for the unaffected leg (− 1.47%, p = 0.001). Both groups responded to the optic flow speed manipulation such that people accelerated with a slow optic flow and decelerated with a fast optic flow. Compared to the semi-immersive GRAIL session, manipulating the optic flow speed with the fully immersive HMD had a greater effect on gait biomechanics whilst also eliciting a higher sense of presence.

**Conclusion:**

Adding fully immersive VR while walking on a self-paced treadmill led to a more cautious gait pattern in people post-stroke. However, walking with the HMD was well tolerated and enjoyable. People post-stroke altered their gait parameters when optic flow speed was manipulated and showed greater alterations with the fully-immersive HMD. Further work is needed to determine the most effective type of optic flow speed manipulation as well as which other principles need to be implemented to positively influence the gait pattern of people post-stroke.

*Trial registration number:* The study was pre-registered at ClinicalTrials.gov (NCT04521829).

**Supplementary Information:**

The online version contains supplementary material available at 10.1186/s12984-023-01254-0.

## Background

In recent years, virtual reality (VR) has been on the rise in the field of healthcare. Over the last 20 years, the popularity and use of VR for physical rehabilitation alone increased remarkably, with increasing evidence supporting its use [1, 2]. However, VR that is used today for physical rehabilitation are often video gaming consoles and were initially designed for entertainment purposes instead of rehabilitation [3]. Consequently, they do not incorporate rehabilitation and motor learning principles to optimally enhance motor rehabilitation. Hence, VR games specifically built for different rehabilitation purposes are required to achieve optimal rehabilitation [3].

One of these rehabilitation purposes that could benefit from VR is post-stroke gait rehabilitation. Post-stroke gait rehabilitation remains a major clinical challenge. Two-thirds of all stroke survivors suffer from walking impairments, causing them to experience a decrease in activities of daily living, level of participation and quality of life [4–6]. People post-stroke often have an asymmetric gait pattern characterized by a shorter stance time and longer swing time of the affected limb and a longer stance time and shorter swing time of the unaffected limb [7]. This asymmetry leads to alterations in step length and a reduced walking speed and cadence [7]. In order to improve these impairments, people post-stroke often receive treadmill training, a repetitive and task-specific gait training that has the potential to enhance neural plasticity—the ability to create permanent structural and functional changes of the brain and spinal cord—which is vital to trigger the learning process of the sensorimotor system [8, 9].

Controlling our locomotion is a complex, multisensory process and involves the integration of visual, vestibular, and proprioceptive information [10]. An important source of visual information used to guide locomotion is optic flow. Optic flow refers to the pattern of visual motion experienced while moving around and is being projected onto the retina of the eye. It provides us with information about the direction and speed of locomotion [10, 11]. During normal walking, the optic flow and proprioceptive information are congruent. However, with the use of VR, the speed of optic flow can be manipulated in such way that there is a mismatch between the optic flow and the proprioceptive information of the lower limbs [12]. As a result, people will adjust their gait pattern in order to diminish this incongruity [13].

Optic flow speed and its influence on locomotion has been examined in the healthy population [14–18] and more specific in older adults [19], but also in several clinical populations, such as neurological patients [13, 20–22]. It is suggested that optic flow can exert an influence on locomotion, but there are conflicting results between populations [10, 13]. In general, it seems that healthy people will increase their walking speed with a slower optic flow and decrease their speed with a faster optic flow [14–16, 18]. This strategy can be altered in patients with neurological diseases due to damage in brain areas involved in the perception and use of optic flow [10]. For example, Schubert and colleagues (2005) found that due to the overreliance on visual information in Parkinson’s disease patients, optic flow speed manipulations led to exaggerated walking speed responses compared to healthy people [20]. On the other hand, the study by Lim et al. reported that cerebral palsy children used an opposite strategy and increased their walking speed with a fast optic flow speed and vice versa [23]. It is assumed that people post-stroke still have the ability to use optic flow information during walking, but alterations are possible and responses can be heterogeneous between individuals, depending on the location of the brain lesion [10]. With the use of VR, the selective manipulation of optic flow could be used to induce desired locomotor changes, such as an increase in walking speed, and therefore has the potential to advance the field of post-stroke gait rehabilitation. However, studies about the effect of optic flow speed on locomotion in people post-stroke are still scarce [13, 22]. Given the potential of optic flow speed manipulation, further exploration is necessary to determine how such manipulation could be useful for rehabilitation purposes. A more in-depth analysis of how optic flow speed influences the gait pattern in people post-stroke is needed.

Two key aspects of VR are immersion and sense of presence. Based on the level of immersion, VR devices and systems can be classified into two categories: (1) Semi-immersive or non-immersive VR systems, who let the user perceive both real world and a part of the virtual environment (e.g. TV-screens, projection screens), and (2) Fully immersive VR systems, who fully integrate the user into the virtual environment, by blocking out perception of the real world (e.g. head-mounted displays (HMD)) [24]. The level of immersion has an impact on the user’s VR experience by influencing the sense of presence (i.e. the feeling of being physically present in the virtual world), with stronger feelings of ‘being physically present’ during exposure in more immersive virtual environments [25, 26]. With semi-immersive VR systems participants are still perceiving the real environment and thus also the real optic flow while walking. Therefore, it is expected that the effect of optic flow speed manipulations on gait will be more limited in a non – or semi-immersive virtual environment, compared to a fully immersive virtual environment. However, to the best of our knowledge, no research has been performed so far on the effect of immersion on optic flow speed manipulations.

For these reasons, the aim of this study was three-fold: (1) to investigate the effect of adding fully immersive VR while walking on a self-paced treadmill on the gait biomechanics, simulator sickness and enjoyment, in people post-stroke and healthy people, (2) to investigate the effect of optic flow speed manipulation (two times faster and two times slower than their comfortable walking speed) on the gait biomechanics, in people post-stroke and healthy people and (3) to investigate the effect of the level of immersion (semi-immersive vs. fully immersive) during walking with different optic flow speeds on the gait biomechanics and level of presence, in people post-stroke and healthy people. We hypothesized that: (1) adding fully immersive VR while walking on a self-paced treadmill will alter the gait biomechanics in both groups, (2) both healthy people and people post-stroke will alter their gait pattern in response to the optic flow speed manipulation and (3) the effect of optic flow speed manipulation and the level of presence will be larger with the fully immersive VR.

## Material and methods

### Study design

An experimental, 2-group, repeated measures single-center trial was conducted in which people post-stroke and healthy people performed two VR-enhanced treadmill walking sessions. Both sessions were identical and carried out on two separate points in time within 10 days, only the VR system used to manipulate the optic flow speed differed: the semi-immersive Gait Real-time Interactive Lab (GRAIL) system and the fully immersive head-mounted display (HMD). The study took place at the Smart Space lab of the University Hospital in Ghent, Belgium. The study was approved by the Ethics Committee of the University of Brussels and the University Hospital of Ghent (B1432020000120) and pre-registered at ClinicalTrials.gov (NCT04521829). The results of this study has been reported in two different papers. The first paper reports on the effect of adding and manipulating optic flow speed in a semi-immersive virtual environment (GRAIL session, paper submitted). The current paper reports on the effect of adding and manipulating optic flow speed in a fully immersive virtual environment (HMD session) and compares the GRAIL and HMD session to investigate the effect of the level of immersion.

### Participants

Chronic, ambulatory stroke patients and age – and sex matched healthy adults were included. The following inclusion criteria were used for the stroke population: (1) diagnosed with stroke (as defined by the World Health Organization), (2) adult (≥ 18 years), (3) stroke onset ≥ 3 months, (4) ambulatory with an impaired gait pattern (Functional Ambulation Categories (FAC) score 2, 3 or 4), (5) ability to walk on a treadmill for 4 times 8 min without bodyweight support, (6) to signal pain, fear and discomfort and (7) to give informed consent. People post-stroke were excluded if they had (1) other neurological deficits leading to impaired gait (e.g. Parkinson’s disease, multiple sclerosis), (2) comorbidities (e.g. COPD, severe osteoporosis, cardiovascular instability), (3) visual and/or vestibular disorders that can interfere with the VR (e.g. Meniere’s disease), (4) severe spasticity of the lower limbs (Modified Ashworth Scale > 2), (5) acute medical illness, (6) the inability to understand and carry out instructions and (7) severe unilateral spatial neglect.

For the healthy participants, the following inclusion criteria were used: (1) normal or corrected-to-normal vision with glasses or contact lenses and (2) no locomotion impairments. Participants were excluded if they (1) have had significant lower extremity injuries during the last two years that might affect their gait and (2) had any type of vestibular/visual deficiency.

Based on a sample size calculation (G*Power 3.1.9.4) (F-tests, repeated measures ANOVA, within-between subjects) with Cohen’s f of 0.25 (moderate effect size), type 1 error probability of 0.05, power of 0.80 for 2 groups and 4 conditions, a minimum of 24 participants, divided equally in 2 groups, had to be recruited.

### Apparatus

Participants walked on the treadmill of the GRAIL system, an integrative motion capture system consisting of 10 optical motion cameras (Vicon Inc., UK), a dual belt treadmill with integrated force sensors, a 180-degree cylindrical projection screen and D-Flow software (Motekforce Link, Netherlands). The treadmill of the GRAIL system has two modes: fixed walking speed or self-paced. For this study, the treadmill was self-paced, meaning that the participants had control over the speed of the treadmill and could start, stop and change speed at will. For safety reasons, participants wore a safety harness and the maximum walking speed was set at 2 m/s.

**Fig. 1:**
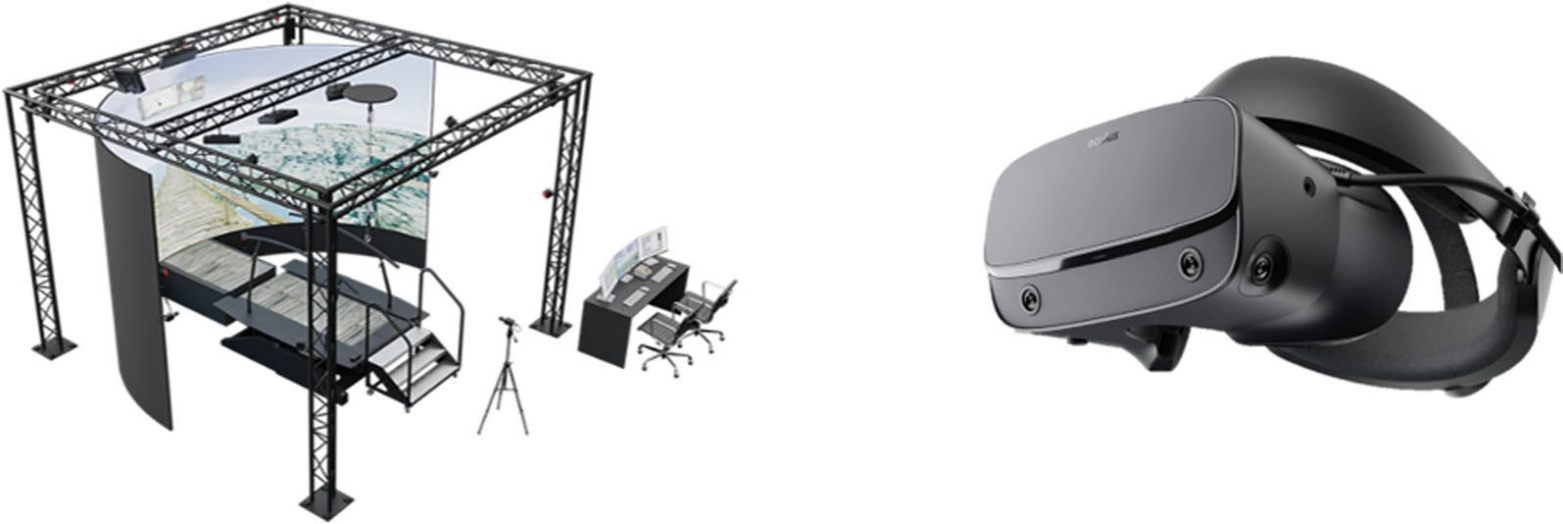
The GRAIL system with the semi-immersive projection screen (left) and with the fully immersive HMD ‘Oculus Rift’ (right)

**Fig. 2:**
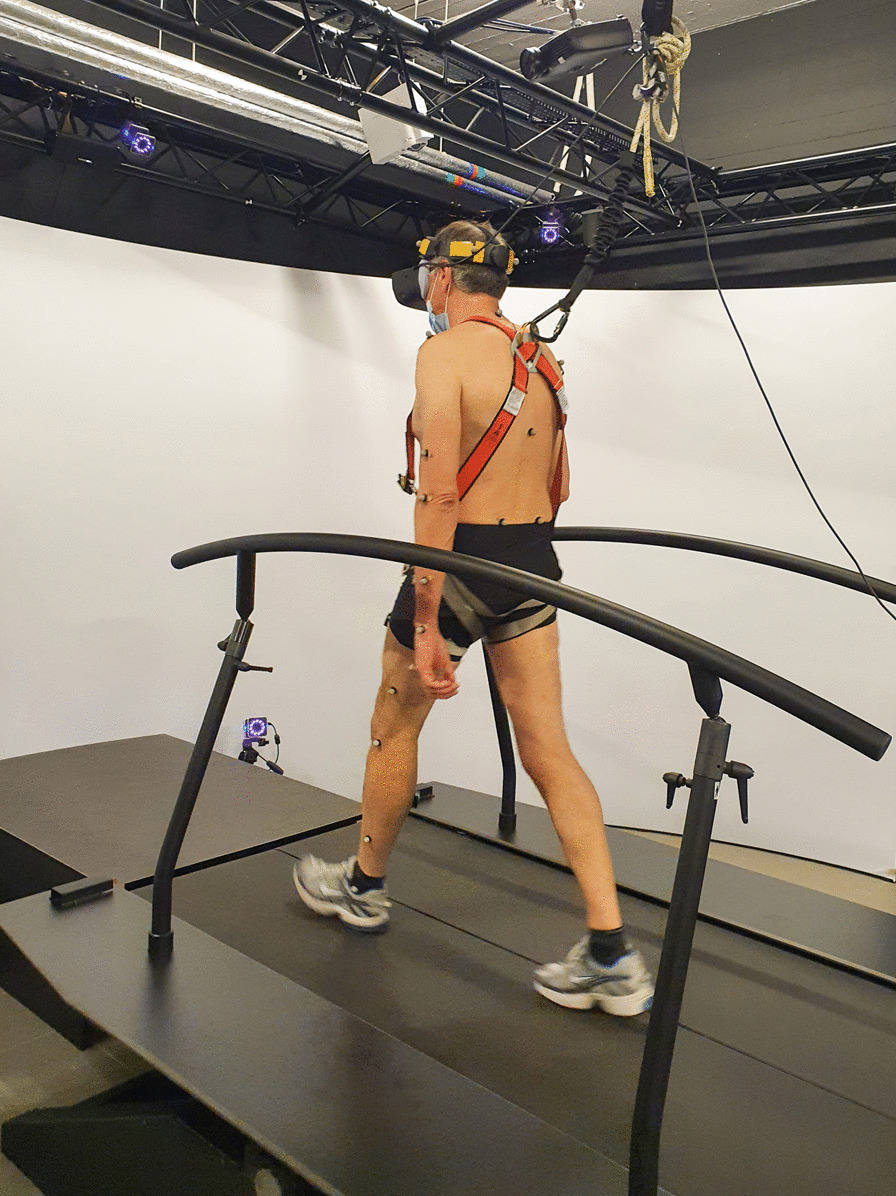
A picture of a participant walking on the GRAIL system with the reflective markers while wearing the HMD

The 180-degree cylindrical projection screen of the GRAIL system provided the semi-immersive VR, while the HMD VR system ‘Oculus Rift’ (Oculus, LLS, US) ensured the fully immersive VR (Figs. 1 and 2). The virtual environment used in this study was a standard environment provided by Motek and represented a city street in the Italian Alps. For this study, the game elements of this environment (i.e. collecting ingredients on the street) were removed and participants only had to walk forward. The same virtual environment was shown in both VR devices.

### Experimental procedure

**Fig. 3:**
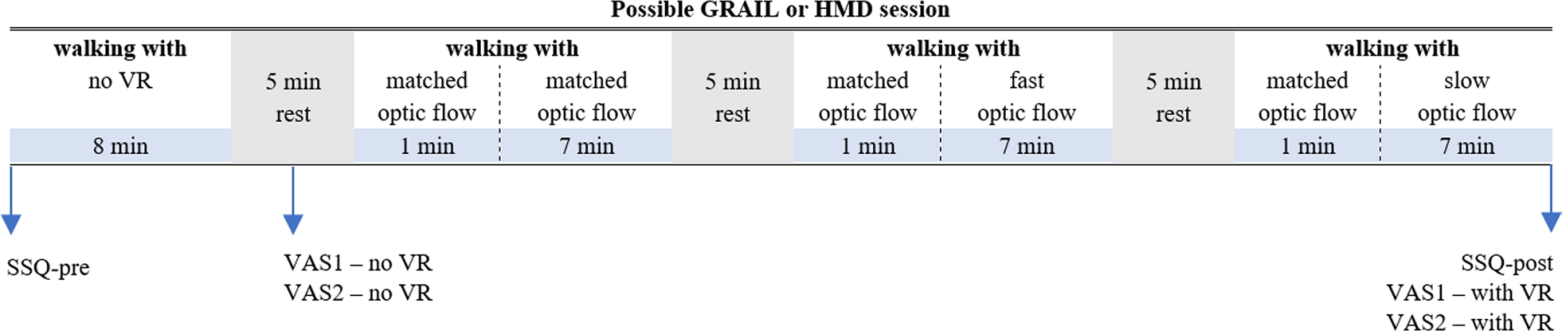
A protocol timeline for a possible GAIL or HMD session

In each session, both groups underwent five walking trials (see Fig. 3 for the protocol timeline). The first trial consisted of 8 min where participants walked without VR to get familiarized with self-paced walking [27]. For the second trial, the habituation trial, the VR was added and participants walked for 5 min with either the large projection screen of the GRAIL system (GRAIL session) or with the VR glasses (HMD session) to get used to walking with VR. Thereafter, participants underwent three more walking trials of 8 min during which the optic flow speed was being manipulated: 2 times slower than, equal to and 2 times faster than their comfortable walking speed. The duration of 8 min was chosen to investigate if and how long the changes that are expected immediately after the manipulation of optic flow maintain. A longer duration was not feasible and/or would evoke fatigue. The comfortable walking speed of the participants was defined as the average walking speed during the 5-min habituation trial. The optic flow speed manipulation occurred after one minute and lasted for the remaining 7 min. The order of the two sessions (GRAIL, HMD) and the optic flow speed manipulation within the session (matched, slow, fast) was randomized through block randomization in Microsoft Excel®. Participants were not informed about this manipulation. In-between walking rounds, participants were given a 5-min rest period to complete a few short questionnaires.

### Outcomes and pre-processing

Our primary outcome measures were lower limb kinematics (i.e. hip, knee and ankle in the sagittal plane) and spatiotemporal gait parameters (i.e. walking speed, cadence, stride time, step length, swing – and stance time, step width).

Kinematic data were recorded with the use of a 10-camera VICON Vero 1.3 system at 100 Hz using the full body Plug-in-Gait model provided by Vicon. In this study we only used marker data from the lower limbs. Sagittal kinematic marker data of the hip, knee and ankle were processed using Vicon Nexus software. Gait cycle segmentation of kinematic data and calculation of the spatiotemporal gait parameters (i.e. cadence, stride time, step length, swing – and stance time, step width) were performed in Python 3.7. (Anaconda Inc., USA) with custom-made scripts. Walking speed was measured continuously and was derived directly from the treadmill system. Data were resampled to 100 Hz with custom-made scripts in Python 3.7.

Our secondary outcome measures were simulator sickness, sense of presence and level of enjoyment. Simulator sickness was assessed with the Simulator Sickness Questionnaire (SSQ) [28]. The SSQ is a widely used questionnaire to evaluate simulator sickness when using VR and consists of 16 symptoms. Before and after the walking trial, participants had to indicate on a four-point Likert scale ranging from 0 (none) to 3 (severe) how much each symptom was affecting them at that moment. The overall score is measured by adding the scores of the 16 items and multiplying the achieved sum by 3.74 [28]. The total score can serve as an indicator of the severity of the simulator sickness and ranges between 0 – 179.52 with higher scores indicating higher levels of simulator sickness.

The sense of presence experienced in a virtual environment was assessed with the Igroup Presence Questionnaire (IPQ) [29]. The IPQ consists of 14 questions divided in three subscales (spatial presence, involvement, experienced realism) and one additional general item not belonging to a subscale. “Spatial presence” measures the sense of being physically present (e.g. “Somehow I felt that the virtual world surrounded me”), “involvement” measures the attention devoted to the virtual environment and involvement experienced (e.g. “How aware were you of the real world surrounding while navigating in the virtual world?”) and “Experienced realism” measures the subjective experience of realism (e.g. “How real did the virtual world seem to you?”). The IPQ is scored on a seven-point Likert scale ranging from 0 (when totally disagreeing with the statement) to 6 (when totally agreeing with the statement). The maximus score of the IPQ in total is 84. The maximum score for the subscales are 6 for the general item, 30 for spatial presence, 24 for involvement and 24 for experienced realism. At the end of each session, participants had to fill in the IPQ.

Lastly, the level of enjoyment was assessed with two Visual Analogue Scales (VAS). After walking without the VR and with the VR, participants were asked to answer following two questions: VAS1 – Indicate on the line below how much you enjoyed walking on the treadmill under these conditions, VAS2 – Indicate on the line below whether you would like to do this type of gait training during your rehabilitation (stroke group only). Participants had to answer these questions by drawing a line on a 10 cm horizontal line. At both ends of the line, opposite answers were provided. Using a ruler, the score can be determined by measuring the distance (cm) on the 10-cm line between the beginning of the line (left side) and the participant’s mark, providing a range of scores from 0–10.

### Statistical analysis

IBM SPSS Statistics version 28, custom-made scripts in Python 3.7. and Matlab (R2022a) were used for statistical analysis. Level of significance was set at α = 0.05. Baseline characteristics between groups were compared using an independent sample t-test and Mann–Whitney U test for respectively normally and not-normally distributed continuous variables and a Chi-squared test for categorical variables.

To investigate the effect of fully immersive VR on the gait biomechanics, the averages during the last 30 s of the trial without VR were compared to those obtained during the last 30 s of the habituation trial. For spatiotemporal data, linear mixed-effect models (LMM) were used. LMM were conducted with condition (no VR, with VR) and group (post-stroke, healthy) as fixed factors, accounting for the within subject correlations and a random intercept of participants. Multiple models were built in SPSS. With the use of the Akaike’s Information Criteria (AIC) value, the best fitted model was chosen (with smaller AIC values indicating a better model). The within subject covariance was unstructured. For kinematic data, statistical parametric mapping (SPM) was used [30]. A SPM two-way Analysis of Variance (ANOVA) was performed to examine the effect of condition (no VR, with VR) and group (post-stroke, healthy): the F-statistic (SPM{F}) was calculated at each point of the time-series. Where SPM{F} crossed a threshold equivalent to α = 0.05, post-hoc Bonferroni analyses were performed using SPM paired t-tests. For post-hoc comparisons, the SPM{t} statistic was calculated for each comparison. The critical threshold was set equivalent to α = 0.0253 to account for multiple comparisons. The t-statistic (SPM{t}) was calculated at each point of the time-series and where SPM{t} crossed the threshold, significant differences were found.

To investigate the effect of optic flow speed on the gait biomechanics, four time points were compared to examine both the short-term and long-term effect: the averages during the 30 s before the manipulation, compared to those obtained during the 30 s immediately after the manipulation, the middle 30 s and the last 30 s of the 8-min trial. LMM were conducted for spatiotemporal parameters, with optic flow condition (matched, fast, slow), time (pre manipulation, post manipulation, middle and end of the trial) and group (post-stroke, healthy) as fixed factors, accounting for the within subject correlations and a random intercept of participants. Multiple models were again built in SPSS and with the use of the AIC the best fitted model was chosen. The within subject covariance was unstructured. For the kinematic data, a SPM two-way repeated measures ANOVA was performed to examine the effect of time (pre manipulation, post manipulation, middle and end of the trial) and group (post-stroke, healthy) in each optic flow condition: the SPM{F} was calculated at each point of the time-series. Where SPM{F} crossed a threshold equivalent to α = 0.05, post-hoc Bonferroni analyses were performed using SPM paired t-tests. For post-hoc comparisons, the SPM{t} statistic was calculated for each comparison. The critical threshold was set equivalent to α = 0.017 to account for multiple comparisons. Significant differences were recorded where the SPM{t} crossed this threshold.

To investigate the effect of the level of immersion during walking with different optic flow speeds on the gait biomechanics, the mean differences (MD) of three comparisons (pre–post manipulation, pre–mid trial, and pre–end trial) of the GRAIL session were compared to those obtained from the HMD session in both groups with a paired sample t-test. For the sense of presence, LMM were conducted with condition (GRAIL, HMD) and group (post-stroke, healthy) as fixed factors, accounting for the within subject correlations. Models were build using the AIC in SPSS. The within subject covariance was unstructured.

## Results

### Subjects characteristics

Sixteen people post-stroke and 16 age—and sex matched healthy controls participated in this study. There were no significant differences in baseline characteristics observed between groups, with exception for the score on the Beck Depression Inventory (BDI). People post-stroke scored significantly higher on this questionnaire (higher total scores indicate more severe depressive symptoms) (Table 1).

**Table 1:** Subjects’ demographic and clinical characteristics

Characteristic	Stroke (n = 16)	Healthy (n = 16)	*p-*value
Age (years)	53.88 ± 11.43	53.75 ± 11.61	0.976
Sex Male (n, %) Female (n, %)	10 (62.5) 6 (37.5)	10 (62.5) 6 (37.5)	1.000
Height (cm)	172.00 ± 8.52	173.50 ± 6.82	0.587
Weight (kg)	74.18 ± 14.35	73.41 ± 11.13	0.867
BDI score	10.88 ± 8.68	1.81 ± 2.46	< 0.001
Time since stroke (months)	44.24 (49.20)	-	
Paretic side Left (n, %) Right (n, %)	9 (56.25) 7 (43.75)	- -	
FAC score	4	-	
Fugl-Meyer lower limb (/34)	22.69 (6.87)	-	

*BDI* Beck Depression Inventory. *FAC* Functional Ambulation Categories. Values are expressed in number (percentage) or mean ± standard deviation

### Effect of fully immersive VR on the gait biomechanics

#### Spatiotemporal gait parameters

The resulting LMM focusing on the effect of condition and group suggested that no significant interaction effect between condition and group was found for all spatiotemporal gait parameters (see Additional file 1: Table S1 for all models). For cadence, stride time, stance time (unaffected leg post-stroke), swing time (unaffected leg post-stroke) and step width a main effect of condition was found (Table 2). When walking with the immersive VR, people post-stroke walked with a significantly slower cadence (MD -3.69strides/min [− 6.22; − 1.15], p = 0.006), a longer stride time (MD 0.10 s [0.02;0.18], p = 0.017) and stance time of the unaffected leg (MD 1.47% [0.61;2.32], p = 0.001) and a shorter swing time of the unaffected leg (MD -1.47% [− 2.32; − 0.61], p = 0.001). The healthy controls significantly reduced their step width when walking with the VR (MD -1.93 cm [− 3.09; − 0.77], p = 0.002).

**Table 2:** Effect of fully immersive virtual reality on the spatiotemporal gait parameters

		No VR	with VR	MD	No VR vs. VR *p value*
Walking speed (m/s)	Stroke	0.88 [0.72;1.04]	0.79 [0.66;0.93]	− 0.09 [− 0.19; 0.01]	0.085
	Healthy	1.39 [1.24;1.55]	1.37 [1.23;1.50]	− 0.02 [− 0.13; 0.08]	0.625
Cadence (strides/min)	Stroke	48.07 [44.04;52.09]	44.38 [40.63;48.14]	− 3.69 [− 6.22; − 1.15]	0.006*
	Healthy	56.51 [52.61;60.41]	55.65 [52.01;59.28]	− 0.86 [− 3.32; 1.59]	0.477
Stride time (sec)	Stroke	1.32 [1.20;1.44]	1.42 [1.30;1.54]	0.10 [0.02; 0.18]	0.017*
	Healthy	1.07 [0.95;1.18]	1.08 [0.97;1.20]	0.02 [− 0.06; 0.10]	0.641
Step length affected^♦^ (cm)	Stroke	50.97 [46.11;55.82]	51.83 [47.37;56.29]	0.86 [− 2.84; 4.57]	0.637
	Healthy	66.79 [62.09;71.49]	67.85 [63.53;72.16]	1.06 [− 2.53; 4.65]	0.551
Step length unaffected^♦^ (cm)	Stroke	49.82 [44.90;54.73]	49.29 [44.60;53.98]	− 0.53 [− 3.98; 2.93]	0.757
	Healthy	66.79 [62.03;71.55]	67.85 [63.31;72.39]	1.06 [− 2.28; 4.41]	0.523
Stance time affected^♦^ (%GC)	Stroke	67.14 [65.62;68.67]	67.98 [66.77;69.18]	0.83 [− 0.26; 1.93]	0.132
	Healthy	65.18 [63.71;66.66]	65.77 [64.61;66.94]	0.59 [− 0.48; 1.65]	0.268
Stance time unaffected^♦^ (%GC)	Stroke	69.85 [68.36;71.35]	71.32 [69.71;72.93]	1.47 [0.61; 2.32]	0.001*
	Healthy	65.18 [63.73;66.63]	65.77 [64.21;67.33]	0.59 [− 0.24; 1.41]	0.156
Swing time affected^♦^ (%GC)	Stroke	32.86 [31.33;34.38]	32.02 [30.82;33.23]	− 0.83 [− 1.93; 0.26]	0.123
	Healthy	34.82 [33.34;36.29]	34.23 [33.06;35.39]	− 0.59 [− 1.65; 0.48]	0.268
Swing time unaffected^♦^ (%GC)	Stroke	30.15 [28.65;31.64]	28.68 [27.07;30.29]	− 1.47 [− 2.32; − 0.61]	0.001*
	Healthy	34.82 [33.37;36.27]	34.23 [32.67;35.79]	− 0.59 [− 1.41; 0.24]	0.156
Step width affected^♦^ (cm)	Stroke	18.08 [16.02;20.14]	17.11 [15.07;19.14]	− 0.97 [− 2.17; 0.23]	0.109
	Healthy	14.03 [12.03;16.03]	12.10 [10.13;14.07]	− 1.93 [− 3.09; − 0.77]	0.002*
Step width unaffected^♦^ (cm)	Stroke	18.07 [15.98;20.16]	16.98 [14.93;19.03]	− 1.09 [− 2.31; 0.14]	0.080
	Healthy	14.03 [12.01;16.05]	12.10 [10.11;14.09]	− 1.93 [− 3.12; − 0.74]	0.002*

Values are reported in mean with 95% confidence interval and MD (mean difference) with 95% confidence interval. *VR* virtual reality, %GC: percentage of gait cycle. ^♦^for the healthy participants the average of the left and right side is used. The asterisk indicates a significant difference

#### Kinematics

SPM two-way ANOVA analyses were performed on 15 subjects in each group, due to missing data of one person in the stroke group (missing data was due to one or more Vicon markers that fell off while walking). To maintain equal group sizes, the healthy matched participant was also removed. The SPM two-way ANOVA revealed no significant interaction effect, nor a main effect of condition (Additional file 1: Figs. S1 and S2).

### Effect of fully immersive VR on simulator sickness and enjoyment

#### Simulator sickness

Only the stroke group had a significant increase in the SSQ after walking with the VR, from 4.68(± 7.03) points to 11.69(± 11.97) points (MD 7.01(± 6.53) points, p = 0.003). The healthy group had a non-significant increase from 1.17(± 2.25) points to 2.10(± 3.04) points (MD 0.94(± 2.55) points, p = 0.157). The difference between groups was significant (p = 0.003).

#### Enjoyment

Both groups indicated that they enjoyed walking on the treadmill with VR more compared to walking on the treadmill without VR as indicated by a significant increase in VAS1 (Table 3). The difference between groups was not significant. The stroke group would also like to implement VR in their gait training as indicated by a higher score on the VAS2 with VR compared to without VR.

**Table 3:** Results of the two Visual Analogue Scales

		No VR	With VR	MD	Within group p-value	Between group p-value
VAS1 (/10)	Stroke	4.99(± 2.01)	6.76(± 2.73)	1.77(± 2.32)	0.010*	0.395
	Healthy	6.11(± 2.23)	7.11(± 2.14)	1.01(± 1.57)	0.005*	
VAS2 (/10)	Stroke	6.47(± 2.49)	7.90(± 1.90)	1.43(± 2.04)	0.015*	–

Values are reported in mean with SD and MD (mean difference) with SD. VAS1: How much did you enjoy walking on the treadmill under these conditions? VAS2: Would you like to do this type of gait training during your rehabilitation (people post-stroke only)? The asterisk indicates a significant difference

### Effect of optic flow speed manipulation on the gait biomechanics

#### Spatiotemporal gait parameters

The resulting LMM focusing on optic flow condition (matched, fast, slow), time (pre manipulation, post manipulation, middle and end of the trial) and group (post-stroke, healthy) suggested interactions between condition and time with a main effect of group for step length, stance and swing time (affected leg post-stroke) and step width (see Additional file 1: Table S2 for all models). A three-way interaction between optic flow condition, time and group was suggested for walking speed, cadence, stride time, stance and swing time (unaffected leg post-stroke).

Table 4 shows the MD between optic flow condition, group and time for all spatiotemporal gait parameters. Significant interaction effects revealed that in both groups, the slow and fast optic flow speed manipulation led to significant changes in several spatiotemporal gait parameters. Immediately after the fast optic flow manipulation, both groups significantly decreased their walking speed (stroke: MD -0.10 m/s [− 0.16;-0.04], p < 0.001; healthy: MD − 0.12 m/s [− 0.18; − 0.06], p < 0.001). This decrease in walking speed was maintained over time in the stroke group only (till mid trial). In the slow optic flow condition, immediately after the manipulation both groups significantly increased their walking speed (stroke: MD 0.06 m/s [0.02;0.10], p < 0.001; healthy: MD 0.07 m/s [0.03;0.10], p < 0.001). This increase in walking speed was only maintained over time in the healthy group.

**Table 4:** Effect of optic flow speed on the spatiotemporal gait parameters

Condition	Group	Time point		Walking speed (m/s)		Cadence (stride/min)		Stride time (s)		Step length (cm)			
				MD [95% CI]	*p-*value	MD [95% CI]	*p-*value	MD [95% CI]	*p-*value	MD [95% CI]	*p-*value	MD [95% CI]	*p-*value
										Affected leg		Unaffected leg	
Matched optic flow	Stroke	Pre	Post	0.03 [0.00;0.06]	.081	0.21 [-0.44;0.87]	1.000	-0.01 [-0.03;0.01]	1.000	1.33 [-0.32;2.97]	.185	1.61 [-0.29;3.51]	.138
			Mid	0.04 [− 0.06;0.14]	1.000	0.56 [− 0.96;2.09]	1.000	− 0.02 [− 0.06;0.02]	1.000	2.02 [− 1.44;5.48]	.662	2.34 [− 1.29;5.97]	.478
			End	− 0.05 [− 0.14;0.04]	.812	− 1.28 [− 2.61;0.05]	.066	0.05 [0.01;0.09]	.011*	0.08 [− 2.64;2.81]	1.000	0.46 [− 2.80;3.73]	1.000
	Healthy	Pre	Post	0.02 [− 0.01;0.05]	.270	0.06 [− 0.60;0.71]	1.000	0.00 [− 0.02;0.02]	1.000	1.16 [− 0.49;2.81]	.335	1.16 [− 0.73;3.06]	.566
			Mid	0.09 [− 0.01;0.19]	.091	1.32 [− 0.21;2.84]	.125	− 0.03 [− 0.07;0.02]	.557	2.67 [− 0.79;6.13]	.227	2.67 [− 0.96;6.30]	.282
			End	0.06 [− 0.03;0.15]	.458	1.01 [− 0.33;2.35]	.247	− 0.02 [− 0.06;0.02]	.916	2.19 [− 0.53;4.92]	.184	2.13 [− 1.14;5.40]	.460
Fast optic flow	Stroke	Pre	Post	− 0.10 [− 0.16;− 0.04]	< .001*	− 2.41 [− 3.58;− 1.23]	< .001*	0.10 [0.04;0.16]	< .001*	− 4.58 [− 7.29;− 1.87]	< .001*	− 5.01 [− 7.99;− 2.02]	< .001*
			Mid	− 0.08 [− 0.16;0.00]	.037*	− 2.12 [− 3.96;− 0.29]	.016*	0.08 [0.01;0.14]	.011*	− 2.13 [− 4.66;0.39]	.142	− 2.50 [− 5.19;0.18]	.079
			End	− 0.07 [− 0.19;0.04]	.512	− 2.57 [− 4.63;− 0.51]	.008*	0.13 [− 0.02;0.29]	.127	− 2.41 [− 6.21;1.38]	.498	− 2.47 [− 6.82;1.88]	.715
	Healthy	Pre	Post	− 0.12 [− 0.18;− 0.06]	< .001*	− 1.41 [− 2.58;− 0.24[	.012*	0.04 [− 0.02;0.10]	.574	− 4.37 [− 7.08;− 1.66]	< .001*	− 4.37 [− 7.35;− 1.39]	.001*
			Mid	− 0.01 [− 0.09;0.07]	1.000	− 0.32 [− 2.18;1.53]	1.000	0.01 [− 0.05;0.07]	1.000	− 0.16 [− 2.73;2.40]	1.000	− 0.11 [− 2.83;2.61]	1.000
			End	− 0.10 [− 0.21;0.02]	.155	− 0.89 [− 2.98;1.21]	1.000	0.02 [− 0.13;0.17]	1.000	− 1.30 [− 5.18;2.58]	1.000	− 1.30 [− 5.72;3.11]	1.000
Slow optic flow	Stroke	Pre	Post	0.06 [0.02;0.10]	< .001*	1.44 [0.66;2.21]	< .001*	− 0.05 [− 0.08;− 0.02]	< .001*	2.27 [0.80;3.74]	< .001*	2.40 [0.61;4.19]	.004*
			Mid	0.08 [0.00;0.15]	.053	1.35 [− 0.40;3.10]	.219	− 0.03 [− 0.11;0.05]	1.000	1.78 [− 0.76;4.33]	.344	1.67 [− 1.66;5.00]	1.000
			End	0.08 [− 0.02;0.18]	.204	1.05 [− 1.26;3.35]	1.000	− 0.03 [− 0.10;0.05]	1.000	1.73 [− 1.70;5.17]	.952	1.93 [− 1.53;5.39]	.734
	Healthy	Pre	Post	0.07 [0.03;0.10]	< .001*	1.15 [0.37;1.93]	.001*	− 0.02 [− 0.05;0.01]	.310	1.69 [0.22;3.16]	.017*	1.69 [− 0.10;3.48]	.074
			Mid	0.15 [0.07;0.23]	< .001*	2.42 [0.70;4.14]	.003*	− 0.05 [− 0.13;0.03]	.621	3.99 [1.49;6.50]	< .001*	3.99 [0.71;7.28]	.011*
			End	0.16 [0.05;0.26]	.001*	2.92 [0.61;5.23]	.007*	− 0.06 [− 0.13,0.02]	.262	4.69 [1.26;8.11]	.001*	4.59 [1.13;8.05]	.005*

Condition	Group	Time point		Stance time (% gait cycle)				Swing time (% gait cycle)				Step width (cm)			
				MD [95% CI]	*p-*value	MD [95% CI]	*p-*value	MD [95% CI]	*p-*value	MD [95% CI]	*p-*value	MD [95% CI]	*p-*value	MD [95% CI]	*p-*value
				Affected leg		Unaffected leg		Affected leg		Unaffected leg		Affected leg		Unaffected leg	
Matched optic flow	Stroke	Pre	Post	− 0.28 [− 0.62;0.07]	.189	− 0.19 [− 0.51;0.13]	.603	0.28 [− 0.07;0.62]	.189	0.19 [− 0.13;0.51]	.603	0.03 [− 0.57;0.64]	1.000	0.00 [− 0.60;0.60]	1.000
			Mid	− 0.43 [− 1.05;0.18]	.336	− 0.40 [− 1.09;0.28]	.652	0.43 [− 0.18;1.05]	.336	0.40 [− 0.28;1.09]	.652	0.33 [− 0.28;0.94]	.812	0.34 [− 0.24;0.92]	.651
			End	0.21 [− 0.52;0.94]	1.000	0.21 [− 0.36;0.77]	1.000	− 0.21 [− 0.94;0.52]	1.000	− 0.21 [− 0.77;0.36]	1.000	0.43 [− 0.27;1.14]	.554	0.36 [− 0.38;1.10]	1.000
	Healthy	Pre	Post	− 0.15 [− 0.50;0.19]	1.000	− 0.15 [− 0.47;0.17]	1.000	0.15 [− 0.19;0.50]	1.000	0.15 [− 0.17;0.47]	1.000	0.06 [− 0.55;0.66]	1.000	0.06 [− 0.54;0.66]	1.000
			Mid	− 0.34 [− 0.96;0.27]	.747	− 0.34 [− 1.03;0.35]	1.000	0.34 [− 0.27;0.96]	.747	0.34 [− 0.35;1.03]	1.000	0.15 [− 0.46;0.76]	1.000	0.15 [− 0.44;0.73]	1.000
			End	− 0.27 [− 1.00;0.46]	1.000	− 0.27 [− 0.84;0.30]	1.000	0.27 [− 0.46;1.00]	1.000	0.27 [− 0.30;0.84]	1.000	0.31 [− 0.39;1.02]	1.000	0.32 [− 0.42;1.06]	1.000
Fast optic flow	Stroke	Pre	Post	0.53 [− 0.67;1.72]	1.000	1.46 [0.57;2.35]	< .001*	− 0.53 [− 1.72;0.67]	1.000	− 1.46 [− 2.35;− 0.57]	< .001*	− 0.59 [− 1.28;0.10]	.132	− 0.56 [− 1.32;0.20]	.285
			Mid	0.83 [0.09;1.56]	.020*	0.83 [0.28;1.37]	< .001*	− 0.83 [− 1.56,− 0.09]	.020*	− 0.83 [− 1.37;− 0.28]	< .001*	− 0.29 [− 0.94;0.36]	1.000	− 0.18 [− 0.86;0.50]	1.000
			End	0.95 [− 0.16;2.05]	.133	1.11 [0.21;2.01]	.010*	− 0.95 [− 2.05;0.16]	.133	− 1.11 [− 2.01;0.21]	.010*	0.06 [− 0.82;0.95]	1.000	0.06 [− 0.80;0.92]	1.000
	Healthy	Pre	Post	0.82 [− 0.38;2.01]	.383	0.82 [− 0.07;1.71]	.088	− 0.82 [− 2.01;0.38]	.383	− 0.82 [− 1.71;0.07]	.088	0.46 [− 0.23;1.15]	.430	0.46 [− 0.30;1.22]	.606
			Mid	0.16 [− 0.59;0.90]	1.000	0.16 [− 0.39;0.71]	1.000	− 0.16 [− 0.90;0.59]	1.000	− 0.16 [− 0.71;0.39]	1.000	0.38 [− 0.27;1.04]	.650	0.39 [− 0.30;1.07]	.746
			End	0.37 [− 0.75;1.49]	1.000	0.39 [− 0.53;1.31]	1.000	-0.37 [− 1.49;0.75]	1.000	− 0.39 [− 1.31;0.53]	1.000	0.60 [− 0.32;1.51]	.452	0.58 [− 0.30.1.74]	.443
Slow optic flow	Stroke	Pre	Post	− 0.36 [− 0.90;0.18]	.421	− 0.84 [− 1.30;− 0.38]	< .001*	0.36 [− 0.18;0.90]	.421	0.84 [0.38;1.30]	< .001*	0.44 [− 0.26;1.14]	.523	0.40 [− 0.28;1.09]	.647
			Mid	− 0.32 [− 1.67;1.03]	1.000	− 0.63 [− 1.44;0.19]	.224	0.32 [− 1.03;1.67]	1.000	0.63 [− 0.19;1.44]	.224	0.21 [− 0.64;1.06]	1.000	0.14 [− 0.68;0.97]	1.000
			End	0.05 [− 1.26;1.37]	1.000	− 0.74 [− 1.53;0.04]	.073	− 0.05 [− 1.37;1.26]	1.000	0.74 [− 0.04;1.53]	.073	0.57 [− 0.02;1.17]	.067	0.61 [0.02;1.21]	.042*
	Healthy	Pre	Post	− 0.33 [− 0.88;0.21]	.552	− 0.33 [− 0.79;0.12]	.294	0.33 [− 0.21;0.88]	.552	0.33 [− 0.12;0.79]	.294	0.09 [− 0.60;0.79]	1.000	0.09 [− 0.59;0.78]	1.000
			Mid	− 0.70 [− 2.04;0.64]	.860	− 0.70 [− 1.51;0.11]	.122	0.70 [− 0.64;2.04]	.860	0.70 [− 0.11;1.51]	.122	0.29 [− 0.55;1.13]	1.000	0.29 [− 0.53;1.10]	1.000
			End	− 0.82 [− 2.13;0.49]	.508	− 0.82 [− 1.60;− 0.03]	.037*	0.82 [− 0.49;2.13]	.508	0.82 [0.03;1.60]	.037*	0.43 [− 0.17;1.02]	.313	0.42 [− 0.18;1.01]	.337

Values are reported in MD (mean difference) and 95%CI (95% confidence interval). The asterisk indicates a significant difference

The decrease in walking speed of the stroke group in the fast optic flow condition was accompanied by a slower cadence, longer stride time, decreased step length of the affected and unaffected leg and an increased stance time and decreased swing time of the unaffected leg. The decrease in walking speed of the healthy group was accompanied by a slower cadence and decreased step length. The increase in walking speed of the stroke group in the slow condition was accompanied by a faster cadence, shorter stride time, increased step length of the affected and unaffected leg and a decreased stance time and increased swing time of the unaffected leg. The increase in walking speed of the healthy group was accompanied by a faster cadence and increased step length.

#### Kinematics

The SPM two-way repeated measures ANOVA was performed on 11 subjects in each group due to missing data (4 healthy participant, 1 stroke patient, missing data was due to one or more Vicon markers that fell off while walking). To maintain equal group sizes, the matched participants were removed from the analyses. The SPM analyses revealed a significant interaction effect in the matched condition for the unaffected ankle and knee joint, in the fast condition for the ankle (both affected and unaffected) and affected hip joint, and in the slow condition for the ankle (both affected and unaffected) and unaffected knee joint. A significant main effect of time was found for the ankle and hip (both affected and unaffected side post-stroke) in the matched and fast condition and for the ankle, knee and hip (both affected and unaffected side post-stroke) in the slow condition (Additional file 1: Fig. S3 through 8). In the post-hoc SPM t-tests, the critical thresholds were only exceeded in the slow condition for the ankle, knee and hip joint in both groups (unaffected side post-stroke) when comparing pre and post manipulation (people post-stroke), pre manipulation and mid trial (healthy group), and pre manipulation and end trial (healthy group).

**Fig. 4:**
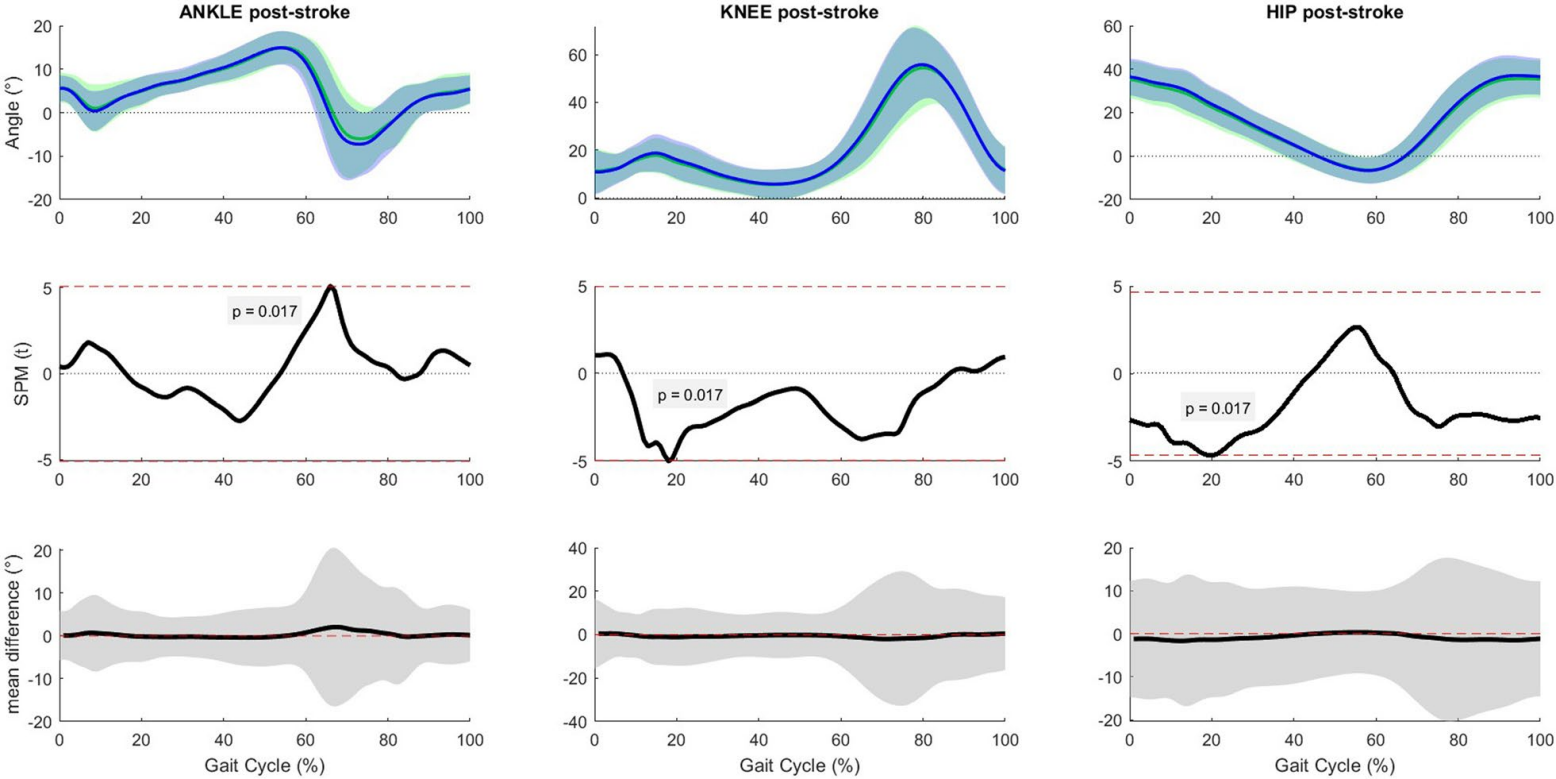
Results of the post-hoc analyses, paired sample t-test for the slow condition (pre vs. post manipulation) in the stroke group (unaffected side). Horizontal axis is percentage gait cycle. First row is mean joint angles ± 1 standard deviation for people post-stroke pre manipulation (green) and post manipulation (blue). Second row shows SPM(t) value throughout the gait cycle. The dashed red line is equivalent to α = 0.02. Third row shows mean difference with 95% confidence interval between pre and post manipulation

**Fig. 5:**
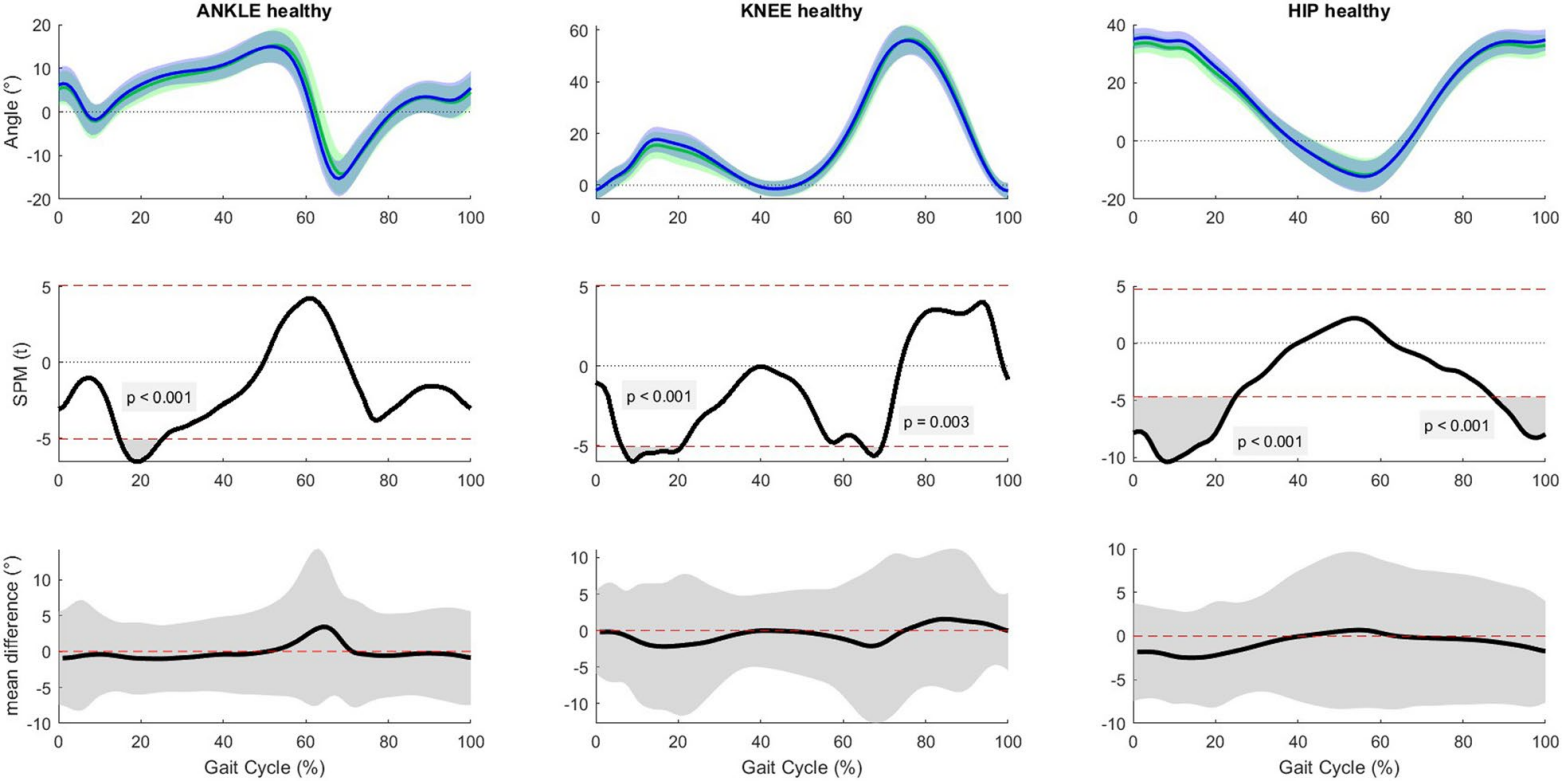
Results of the post-hoc analyses, paired sample t-test for the slow condition (pre manipulation vs. mid trial) in the healthy group. Horizontal axis is percentage gait cycle. First row is mean joint angles ± 1 standard deviation for heathy people pre manipulation (green) and mid trial (blue). Second row shows SPM(t) value throughout the gait cycle. The dashed red line is equivalent to α = 0.02. Third row shows mean difference with 95% confidence interval between pre manipulation and mid trial

**Fig. 6:**
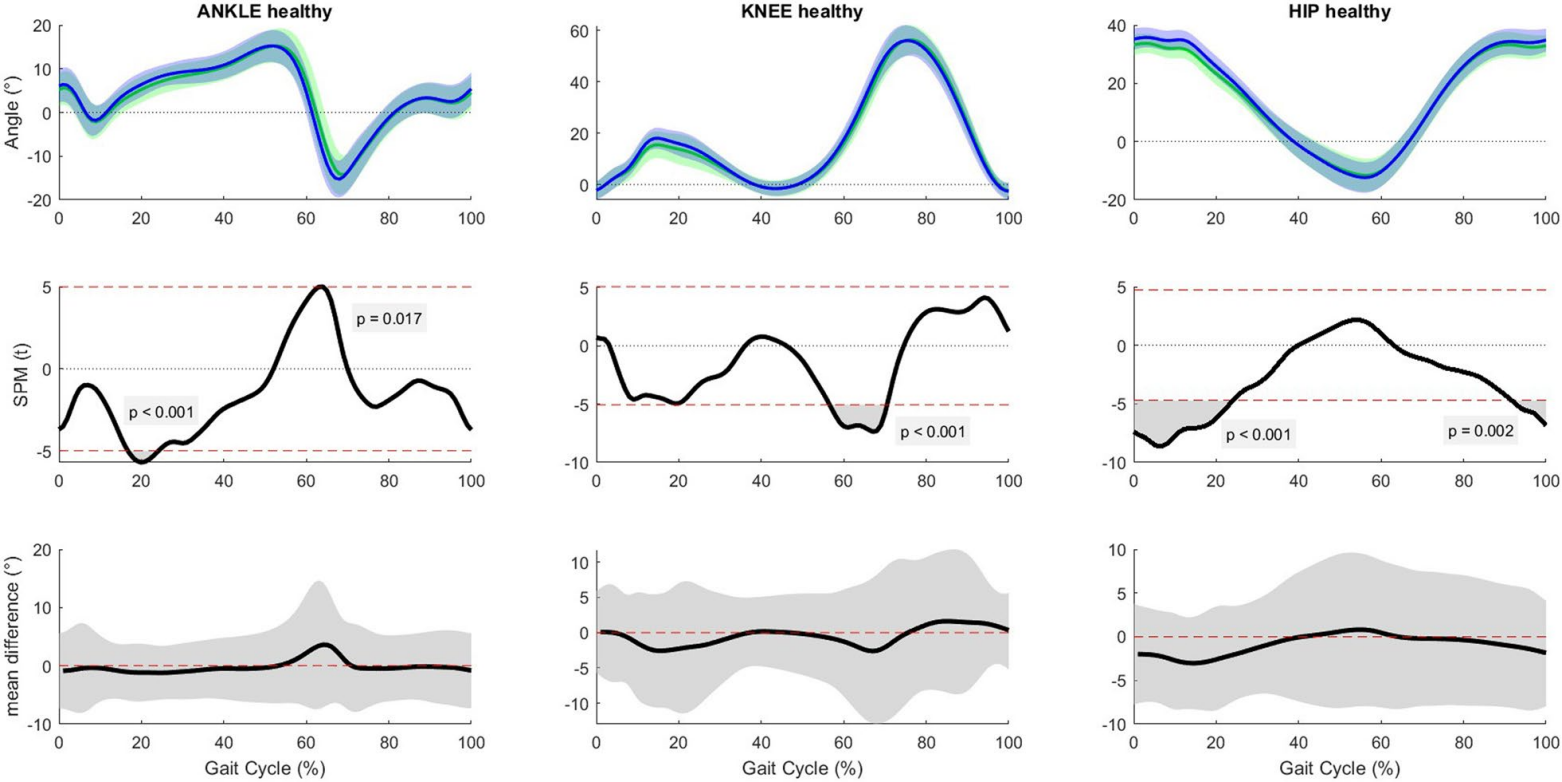
Results of the post-hoc analyses, paired sample t-test for the slow condition (pre manipulation vs. end trial) in the healthy group. Horizontal axis is percentage gait cycle. First row is mean joint angles ± 1 standard deviation for heathy people pre manipulation (green) and end trial (blue). Second row shows SPM(t) value throughout the gait cycle. The dashed red line is equivalent to α = 0.02. Third row shows mean difference with 95% confidence interval between pre manipulation and end trial

Immediately after the slow OF manipulation, people post-stroke had an increase in plantar flexion of 1.99° at 66% of the gait cycle, an increase in knee flexion of 1.07° at 18% of the gait cycle, and an increase in hip flexion of 1.39° at 20% of the gait cycle (Fig. 4). At mid trial, healthy people had an increase in dorsiflexion with a maximum of 1.02° between 15 and 25% of the gait cycle, an increase in knee flexion with a maximum of 2.18° between 6 and 21% and 65–69% of the gait cycle, and an increase in hip flexion with a maximum of 2.48° between 0 and 25% and 87–100% of the gait cycle (Fig. 5). At the end of the trial, healthy people had an increase in dorsiflexion with a maximum of 3.62° between 17 and 24% and at 64% of the gait cycle, an increase in knee flexion with a maximum of 2.61° between 56 – 70% of the gait cycle, and an increase in hip flexion with a maximum of 3.03° between 0 and 24% and 92–100% of the gait cycle (Fig. 6).

### Effect of level of immersion during walking with different optic flow speeds

#### Gait biomechanics

The effect of the level of immersion on the gait biomechanics was only investigated in the spatiotemporal gait parameters since optic flow speed had no or only a very limited effect on the lower limb kinematics. In both sessions, the kinematic changes were very small and did not reach the minimal clinical important difference and are therefore not considered clinically relevant [31, 32].

Table 5 shows the MD between the fast and slow optic flow condition, group and time for both sessions for the most relevant spatiotemporal gait parameters. Both in people post-stroke and healthy people, manipulating the optic flow speed in the fully immersive HMD had a greater effect on spatiotemporal gait parameters compared to the semi-immersive GRAIL system. The most prominent differences were found for walking speed and step length (both affected and unaffected leg post-stroke). With the slow optic flow speed, both groups increased their walking speed and step length more with the fully immersive HMD compared to the semi-immersive GRAIL system. The opposite was seen with the fast optic flow speed, where both groups decreased there walking speed and step length more when the optic flow speed was manipulated with the fully immersive HMD. Results for all optic flow conditions and all spatiotemporal gait parameters can be found in Additional file 1: Table S3.

**Table 5:** Effect of the level of immersion on the spatiotemporal gait parameters

Gait parameter	Group	Condition	Time point		N	GRAIL MD (SD)	HMD MD (SD)	GRAIL vs. HMD *p-value*
Walking speed (m/s)	Stroke	Fast	Pre	Post	16	− 0.01 (0.08)	− 0.10 (0.07)	0.001*
				Mid	16	− 0.01 (0.11)	− 0.08 (0.14)	0.058
				End	16	− 0.01 (0.12)	− 0.07 (0.12)	0.018*
		Slow	Pre	Post	16	0.00 (0.05)	0.06 (0.05)	0.005*
				Mid	16	0.00 (0.06)	0.08 (0.13)	0.087
				End	16	0.01 (0.04)	0.08 (0.15)	0.088
	Healthy	Fast	Pre	Post	16	− 0.04 (0.03)	− 0.12 (0.10)	0.013*
				Mid	16	0.01 (0.07)	− 0.01 (0.09)	0.410
				End	16	0.01 (0.08)	− 0.10 (0.21)	0.043*
		Slow	Pre	Post	16	− 0.01 (0.03)	0.07 (0.06)	< 0.001*
				Mid	16	0.01 (0.04)	0.15 (0.09)	< 0.001*
				End	16	0.01 (0.03)	0.18 (0.11)	< 0.001*
Cadence (stride/min)	Stroke	Fast	Pre	Post	16	− 1.02 (1.59)	− 2.41 (1.79)	0.841
				Mid	16	− 0.88 (2.55)	− 2.12 (3.32)	0.051
				End	15	− 1.07 (2.61)	− 2.66 (3.74)	0.001*
		Slow	Pre	Post	16	1.18 (1.27)	1.44 (1.40)	0.377
				Mid	15	1.10 (2.81)	1.13 (3.00)	0.393
				End	14	1.23 (2.58)	0.66 (4.50)	0.077
	Healthy	Fast	Pre	Post	16	− 0.38 (0.44)	− 1.41 (1.85)	0.030*
				Mid	15	− 0.03 (0.95)	− 0.41 (1.85)	0.302
				End	13	− 0.04 (1.08)	− 0.76 (2.29)	0.143
		Slow	Pre	Post	15	0.65 (0.63)	1.16 (0.83)	0.068
				Mid	15	0.88 (0.93)	2.35 (1.80)	0.008*
				End	14	1.13 (1.61)	2.93 (2.26)	0.017*
Stride time (s)	Stroke	Fast	Pre	Post	16	0.03 (0.05)	0.10 (0.11)	0.030*
				Mid	16	0.03 (0.09)	0.08 (0.12)	0.083
				End	15	0.03 (0.10)	0.14 (0.32)	0.127
		Slow	Pre	Post	16	− 0.04 (0.12)	− 0.05 (0.06)	0.581
				Mid	15	− 0.04 (0.12)	− 0.01 (0.15)	0.642
				End	14	− 0.05 (0.11)	− 0.02 (0.16)	0.446
	Healthy	Fast	Pre	Post	16	0.01 (0.01)	0.04 (0.05)	0.050
				Mid	15	0.00 (0.02)	0.01 (0.04)	0.219
				End	13	0.00 (0.02)	0.02 (0.05)	0.107
		Slow	Pre	Post	15	− 0.01 (0.01)	− 0.02 (0.02)	0.030*
				Mid	15	− 0.02 (0.02)	− 0.05 (0.04)	0.009*
				End	14	− 0.02 (0.03)	− 0.06 (0.05)	0.020*
Step length (cm) *Affected leg*	Stroke	Fast	Pre	Post	16	0.43 (3.55)	− 4.58 (4.30)	< 0.001*
				Mid	16	1.20 (5.17)	− 2.13 (4.46)	0.030*
				End	15	2.36 (5.54)	− 2.60 (6.55)	0.006*
		Slow	Pre	Post	16	2.09 (1.71)	2.27 (2.03)	0.813
				Mid	15	2.68 (5.21)	1.93 (4.71)	0.729
				End	13	2.51 (3.77)	2.89 (5.74)	0.827
Step length (cm) *Unaffected leg*	Stroke	Fast	Pre	Post	16	0.42 (3.84)	− 5.01 (5.02)	0.001*
				Mid	16	− 0.02 (4.99)	− 2.50 (4.84)	0.099
				End	15	0.57 (7.47)	− 2.71 (8.01)	0.141
		Slow	Pre	Post	16	1.79 (3.57)	2.40 (2.94)	0.646
				Mid	15	3.10 (9.15)	1.95 (6.43)	0.726
				End	13	3.23 (8.39)	3.12 (5.21)	0.967
	Healthy	Fast	Pre	Post	16	− 1.55 (1.35)	− 4.37 (3.63)	0.011*
				Mid	15	0.36 (2.98)	− 0.16 (2.75)	0.552
				End	14	0.69 (2.91)	− 1.74 (4.54)	0.039*
		Slow	Pre	Post	15	1.40 (1.52)	1.74 (2.35)	0.616
				Mid	15	1.84 (2.12)	4.09 (2.40)	0.010*
				End	14	1.57 (1.77)	5.14 (2.72)	< 0.001*

Values are reported in MD (mean difference) and SD (standard deviation). *GRAIL* Gait Real-time Interactive Lab, *HMD* head-mounted display. The asterisk indicates a significant difference

#### Sense of presence

Table 6 shows the mean and SD of the IPQ subscales for each group and condition. Additional file 1: Table S4 shows the results of the LMM. The LMM for the *general item* only revealed a main effect of condition and suggested that in both groups, the fully immersive HMD resulted in a significantly higher feeling of being there than the semi-immersive GRAIL screen (MD 0.92 points, p = 0.004). The LMM for the subscales *spatial presence* and *involvement* also revealed a significant main effect of condition and suggested that in both groups, the HMD resulted in a significantly higher spatial presence (MD 0.58 points, p < 0.001) and higher involvement (MD 0.98 points, p < 0.001). The LMM for the subscale *experienced realism* revealed no main effects.

**Table 6:** Results of the igroup presence questionnaire

IPQ subscale	Condition	Stroke	Healthy	GRAIL vs. HMD
		Mean (SD)	Mean (SD)	p-value
General item	GRAIL	3.27 (1.39)	2.94 (1.77)	0.004
	HMD	3.94 (1.48)	4.06 (1.34)	
Spatial presence	GRAIL	3.16 (0.62)	3.09 (0.80)	< 0.001
	HMD	4.00 (0.54)	3.46 (0.75)	
Involvement	GRAIL	2.70 (1.62)	2.50 (1.43)	< 0.001
	HMD	3.78 (1.39)	3.36 (1.18)	
Realness	GRAIL	2.35 (1.34)	1.86 (1.01)	0.053
	HMD	2.56 (1.44)	2.26 (1.15)	

Values are reported in mean and SD (standard deviation). IPQ: Igroup Presence Questionnaire, GRAIL: Gait Real-time Interactive Lab, HMD: head-mounted display

## Discussion

### Walking with immersive VR

Results of this study demonstrated that immersive VR-enhanced treadmill walking with the use of a HMD was accepted by people post-stroke and healthy people. All participants were able to complete all walking trials without having any signs of severe simulator sickness, as indicated by the low total scores on the SSQ in both groups. People post-stroke also reported that they liked walking with the VR more than without and would like to implement VR-enhanced treadmill walking in their gait rehabilitation. These results are in line with recent studies examining the potential of immersive VR for the rehabilitation of neurological patients [33, 34].

The recent study of Winter et al. (2021) reported an increase in walking speed when walking with fully immersive VR compared to walking with no VR in healthy individuals, individuals post-stroke, and individuals with multiple sclerosis [33]. Participants walked in a virtual environment that was designed to increase their motivation during training and consisted of an engaging storyline with the implementation of gamification elements (i.e. rebuilding a virtual world by walking on a path). This is in contrast with the virtual environment used in our study, where participants walked forward in an endless city street with no gaming elements. We found that when people post-stroke walked with the HMD, they walked with a slower cadence, a longer stride time, and a longer stance and shorter swing time of the unaffected leg compared to walking without VR. This contrasts with the increased walking speed reported in the study of Winter et al. (2021). This might be explained by the difference in the type of virtual environment (engaging storyline with gaming elements versus an endless city street without gaming elements), but perhaps also by the difference in the type of treadmill system. In the study of Winter et al. the speed was changed manually by the participants with the use of buttons on the handles. This difference in regulation (top-down versus bottom-up) may result in different responses. Lastly, it must also be noted that prior to this study, patients were not familiar with fully immersive VR-enhanced treadmill walking and changes in spatiotemporal gait parameters may be attributed to a more cautious gait pattern. It is very likely that this more cautious gait pattern will diminish when patients are more acquainted with the HMD. Nonetheless, these results highlight the need to incorporate valuable principles (such as performance feedback, gaming elements or competition) in the virtual environment to influence a person’s gait pattern. More research about implementing such valuable principles in the virtual environment that could positively influence the gait pattern of people post-stroke and could be used during gait training is needed.

### Optic flow speed manipulation

Both groups responded to the optic flow speed manipulation by adjusting their spatiotemporal gait parameters. However, relatively small changes in spatiotemporal gait parameters were reported. The changes in the lower limb joint kinematics were too small to be of any clinical value [31, 32]. Both people post-stroke and healthy controls increased their walking speed with a slow optic flow speed and decreased their walking speed with a fast optic flow speed. However, only the decrease in walking speed with the fast optic flow speed reached the minimal clinically important difference of 0.10 m/s [35]. Improving patients’ walking speed is an important therapeutic outcome and is often a goal of post-stroke rehabilitation [6]. The fact that also the stroke group responded to the optic flow speed manipulation and showed alterations in their gait pattern, provides a rationale to incorporate such manipulations in a VR-enhanced training to promote faster walking speeds. The increased walking speed in people post-stroke was accompanied by a faster cadence and longer step length of both the affected and unaffected leg. However, it must be noted that the increase in walking speed was only maintained over time by the healthy group and not by the people post-stroke. This may indicate that in people post-stroke, the effect of a single manipulation is rather short lasting and may not be sufficient to influence their locomotion. Therefore, it is advisable to further explore the effect of different types of optic flow speed manipulations, such as multiple intermittent manipulations of a constant optic flow speed over a longer period. Before optic flow speed manipulations can be implemented in such a training, further work is needed to determine the most optimal type of optic flow speed manipulation as well as to investigate the carry-over effects to overground walking.

The choice for a constant one-time speed manipulation was based on existing literature [14, 15, 17, 18]. Based on the results of the study of Lamontagne et al. (2007), it is suggested that for people post-stroke constant optic flow speeds are easier to perceive and to integrate than continuously changing optic flow speeds (e.g. sinusoidally patterns of optic flow speed) and could therefore elicit a greater effect on patient’s gait pattern [13]. The one-time manipulation in our study lasted for 7 min, which was longer than most previous studies investigating constant optic flow speed manipulations [15, 17, 18]. Our results stipulated that in people post-stroke, there were mainly changes in spatiotemporal gait parameters immediately after the manipulation, by mid or end trial (respectively 3 or 6 min after the manipulation) these changes were no longer visible. It is therefore suggested that the effect of a one-time optic flow speed manipulation is rather short lasting. More research about different types of manipulations, such as multiple intermittent manipulations of a constant optic flow speed over a longer period of time, is therefore needed.

Other factors that could have influenced the current results and should be investigated in future studies are stroke severity, stroke onset and stroke location. People post-stroke included in this study were all chronic, ambulatory stroke patients who could walk independently, but still experienced some difficulties with stairs or uneven surfaces. The average time post-stroke was 44.24 months but ranged from 3.4 months to 202.5 months (16.8 years). An important limitation of our study is that we did not include the stroke location as a patient characteristic. There is a complex cortical network that is responsible for the perception and use of optic flow during locomotion and involves several visual, multisensory and vestibular areas [36]. When the stroke is located in one of these brain areas, the perception and use of optic flow can be affected and patients could react differently on the optic flow speed manipulations [10]. It is therefore advisable for future research to include specific information about the stroke location as a patient characteristic.

### Semi-immersive vs. fully immersive VR

This is the first study to examine the effect of immersion on sense of presence and during walking with different optic flow speeds by providing a direct comparison of manipulating the optic flow speed in a semi-immersive (GRAIL) and fully immersive (HMD) virtual environment. As hypothesized, both groups reported a higher sense of presence when the virtual environment was presented via the HMD, compared to the semi-immersive GRAIL projection screen. Manipulating the optic flow speed in the fully immersive virtual environment also had a larger effect on the spatiotemporal gait parameters compared to the semi-immersive virtual environment. While walking on the self-paced treadmill with the semi-immersive GRAIL projection screen, participants were still aware of their real environment and thus also of the real optic flow. Furthermore, during the GRAIL session, people also had more visual information because they could look down while walking. It was only during the HMD session that participants were completely immersed in the virtual environment. It is likely that the optic flow speed manipulations were much more noticeable for the participants when walking with the HMD, which could explain its greater effect on locomotion. These results are promising and support the use of more immersive VR devices for rehabilitation.

This study is an initial step to establish fully immersive VR-enhanced treadmill training for people post-stroke. Fully immersive VR devices are still not widely used for rehabilitation today, despite their advantages over less immersive VR systems. Compared to the GRAIL system, the HMD has some important assets: the HMD is a much more affordable system and requires much less space when combined with treadmill walking, making the HMD more suitable to implement in rehabilitation – or even home settings. To date, limited studies investigated long-term fully immersive VR interventions. Therefore, in the future it will be important to also investigate the long-term effect of fully immersive VR interventions for stroke rehabilitation.

This study also included some limitations. The first limitation is the limited inventoried patients’ characteristics included in the study. An important characteristic that was not included as a baseline characteristic is participants’ degree of visual dependency. Controlling our locomotion is a complex task and involves the integration of visual, vestibular, and proprioceptive information. People do not always rely equally on these three sources of information to control their locomotion. For example, it is possible that people have changed their weighting of vision and become more or less visual dependent which could influence their response to optic flow speed manipulations. A second limitation is related to the specific inclusion criteria. Therefore, results apply only to the population studied and are not generalizable to all people post-stroke. Lastly, to investigate the effect of patients’ characteristics that could influence the perception of the optic flow (such as visual dependency), a larger sample size is needed.

## Conclusion

This study demonstrated that adding fully immersive VR while walking on a self-paced treadmill influenced the gait pattern of people post-stroke and led to a slightly more cautious gait pattern. However, walking with the HMD was well tolerated and enjoyable. Manipulating the optic flow speed in a fully immersive virtual environment mainly influenced the spatiotemporal gait parameters of people post-stroke and healthy people. A negative relationship between optic flow speed and walking speed was observed in both groups, meaning that people walked faster with a slower optic flow speed and slower with a faster optic flow speed. Manipulating the optic flow speed in a fully immersive virtual environment had a greater effect on spatiotemporal gait parameters compared to the semi-immersive virtual environment and elicited a greater sense of presence. Further work is needed to determine the most optimal type of optic flow speed manipulation as well as which other principles need to be implemented to positively influence the gait pattern of people post-stroke.

### Supplementary Information

**Additional file 1: Table S1.** Linear mixed models for all spatiotemporal gait parameters – effect VR. **Table S2.** Linear mixed models for all spatiotemporal gait parameters – effect optic flow speed. **Table S3.** Effect of level of immersion on the spatiotemporal gait parameters. **Table S4.** Linear mixed models for the Igroup Presence Questionnaire. **Figure S1.** 2-way ANOVA SPM analyses for the joint angles (affected side post-stroke). **Figure S2.** 2-way ANOVA SPM analyses for the joint angles (unaffected side post-stroke). **Figure S3.** 2-way repeated measures ANOVA SPM analyses matched condition (affected side post stroke). **Figure S4.** 2-way repeated measures ANOVA SPM analyses fast condition (affected side post stroke). **Figure S5.** 2-way repeated measures ANOVA SPM analyses slow condition (affected side post stroke). Figure S6: 2-way repeated measures ANOVA SPM analyses matched condition (unaffected side post stroke). **Figure S7.** 2-way repeated measures ANOVA SPM analyses fast condition (unaffected side post stroke). **Figure S8.** 2-way repeated measures ANOVA SPM analyses slow condition (unaffected side post stroke)

## Data Availability

The datasets generated and/or analyzed during the current study are available from the corresponding author on reasonable request.

## Abbreviations

VR: Virtual reality
HMD: Head-mounted display
GRAIL: Gait Real-time Interactive Lab
SSQ: Simulator Sickness Questionnaire
IPQ: Igroup Presence Questionnaire
VAS: Visual Analogue Scale
LMM: Linear mixed-effect models
AIC: Akaike’s Information Criteria
SPM: Statistical parametric mapping
MD: Mean difference
BDI: Beck Depression Inventory
FAC: Functional Ambulation Categories
