# Supplementary material for: Virtual reality—enhanced walking in people post-stroke: effect of optic flow speed and level of immersion on the gait biomechanics
Source: J Neuroeng Rehabil. 2023 Sep 25;20:124. doi: 10.1186/s12984-023-01254-0 (PMC10518929; doi:10.1186/s12984-023-01254-0)
Supplement: Supplementary file 1 — Additional file 1: Table S1. Linear mixed models for all spatiotemporal gait parameters – effect VR. Table S2. Linear mixed models for all spatiotemporal gait parameters – effect optic flow speed. Table S3. Effect of level of immersion on the spatiotemporal gait parameters. Table S4. Linear mixed models for the Igroup Presence Questionnaire. Figure S1. 2-way ANOVA SPM analyses for the joint angles (affected side post-stroke). Figure S2. 2-way ANOVA SPM analyses for the joint angles (unaffected side post-stroke). Figure S3. 2-way repeated measures ANOVA SPM analyses matched condition (affected side post stroke). Figure S4. 2-way repeated measures ANOVA SPM analyses fast condition (affected side post stroke). Figure S5. 2-way repeated measures ANOVA SPM analyses slow condition (affected side post stroke). Figure S6: 2-way repeated measures ANOVA SPM analyses matched condition (unaffected side post stroke). Figure S7. 2-way repeated measures ANOVA SPM analyses fast condition (unaffected side post stroke). Figure S8. 2-way repeated measures ANOVA SPM analyses slow condition (unaffected side post stroke) [file 12984_2023_1254_MOESM1_ESM.docx]

# Additional files

**Additional table 1: Linear mixed models for all spatiotemporal gait parameters – effect VR**

| Spatiotemporal gait parameter | LMM | Fixed factor(s) | AIC |
| --- | --- | --- | --- |
| Walking speed | 1 | Condition | 28.16 |
|  | 2 | Condition * Group | 7.10 |
| Cadence | 1 | Condition | 418.74 |
|  | 2 | Condition * Group | 407.46 |
| Stride time | 1 | Condition | -11.13 |
|  | 2 | Condition * Group | -20.40 |
| Step length *(affected)* | 1 | Condition | 457.48 |
|  | 2 | Condition * Group | 440.36 |
| Step length *(unaffected)* | 1 | Condition | 458.57 |
|  | 2 | Condition * Group | 439.19 |
| Stance time *(affected)* | 1 | Condition | 288.76 |
|  | 2 | Condition * Group | 286.27 |
| Stance time *(unaffected)* | 1 | Condition | 298.65 |
|  | 2 | Condition * Group | 283.90 |
| Swing time *(affected)* | 1 | Condition | 288.76 |
|  | 2 | Condition * Group | 286.27 |
| Swing time *(unaffected)* | 1 | Condition | 298.65 |
|  | 2 | Condition * Group | 283.90 |
| Step width *(affected)* | 1 | Condition | 329.25 |
|  | 2 | Condition * Group | 322.36 |
| Step width *(unaffected)* | 1 | Condition | 330.57 |
|  | 2 | Condition * Group | 324.31 |

LMM: Linear Mixed-Effects Models. AIC: Akaike’s Information Criterion (smaller values indicate a better model).

**Additional table 2: Linear mixed models for all spatiotemporal gait parameters – effect optic flow speed**

| Spatiotemporal gait parameter | LMM | Fixed factor(s) | AIC |
| --- | --- | --- | --- |
| Walking speed | 1 | Condition * Timepoints | -552.43 |
|  | 2 | Condition * Timepoints + Group | -574.84 |
|  | 3 | Condition * Timepoints * Group | -577.88 |
| Cadence | 1 | Condition * Timepoints | 1683.69 |
|  | 2 | Condition * Timepoints + Group | 1673.29 |
|  | 3 | Condition * Timepoints * Group | 1671.77 |
| Stride time | 1 | Condition * Timepoints | -867.02 |
|  | 2 | Condition * Timepoints + Group | -869.61 |
|  | 3 | Condition * Timepoints * Group | -873.36 |
| Step length *(affected)* | 1 | Condition * Timepoints | 2110.84 |
|  | 2 | Condition * Timepoints + Group | 2080.81 |
|  | 3 | Condition * Timepoints * Group | 2089.11 |
| Step length *(unaffected)* | 1 | Condition * Timepoints | 2139.94 |
|  | 2 | Condition * Timepoints + Group | 2111.48 |
|  | 3 | Condition * Timepoints * Group | 2117.19 |
| Stance time *(affected)* | 1 | Condition * Timepoints | 1107.62 |
|  | 2 | Condition * Timepoints + Group | 1101.62 |
|  | 3 | Condition * Timepoints * Group | 1107.39 |
| Stance time *(unaffected)* | 1 | Condition * Timepoints | 1001.30 |
|  | 2 | Condition * Timepoints + Group | 992.66 |
|  | 3 | Condition * Timepoints * Group | 991.59 |
| Swing time *(affected)* | 1 | Condition * Timepoints | 1107.62 |
|  | 2 | Condition * Timepoints + Group | 1101.62 |
|  | 3 | Condition * Timepoints * Group | 1107.39 |
| Swing time *(unaffected)* | 1 | Condition * Timepoints | 1001.30 |
|  | 2 | Condition * Timepoints + Group | 992.66 |
|  | 3 | Condition * Timepoints * Group | 991.59 |
| Step width *(affected)* | 1 | Condition * Timepoints | 1117.60 |
|  | 2 | Condition * Timepoints + Group | 1110.68 |
|  | 3 | Condition * Timepoints * Group | 1118.11 |
| Step width *(unaffected)* | 1 | Condition * Timepoints | 1117.76 |
|  | 2 | Condition * Timepoints + Group | 1110.90 |
|  | 3 | Condition * Timepoints * Group | 1119.97 |

LMM: Linear Mixed-Effects Models. AIC: Akaike’s Information Criterion (smaller values indicate a better model).

**Additional table 3: Effect of level of immersion on the spatiotemporal gait parameters.**

| Gait parameter | Condition | Group | Time point | | N | GRAIL session  MD (SD) | HMD session  MD (SD) | GRAIL vs. HMD  p-value |
| --- | --- | --- | --- | --- | --- | --- | --- | --- |
| Walking speed (m/s) | Matched | Stroke | pre | post | 16 | 0.01 (0.05) | 0.03 (0.04) | *.189* |
|  |  |  |  | mid | 16 | 0.02 (0.08) | 0.04 (0.06) | *.219* |
|  |  |  |  | end | 16 | 0.05 (0.10) | -0.05 (0.14) | *.008** |
|  |  | Healthy | pre | post | 16 | 0.01 (0.04) | 0.02 (0.05) | *.409* |
|  |  |  |  | mid | 16 | 0.03 (0.08) | 0.09 (0.20) | *.323* |
|  |  |  |  | end | 16 | 0.05 (0.07) | 0.06 (0.12) | *.922* |
|  | Fast | Stroke | pre | post | 16 | -0.01 (0.08) | -0.10 (0.07) | *.001** |
|  |  |  |  | mid | 16 | -0.01 (0.11) | -0.08 (0.14) | *.058* |
|  |  |  |  | end | 16 | -0.01 (0.12) | -0.07 (0.12) | *.018** |
|  |  | Healthy | pre | post | 16 | -0.04 (0.03) | -0.12 (0.10) | *.013** |
|  |  |  |  | mid | 16 | 0.01 (0.07) | -0.01 (0.09) | *.410* |
|  |  |  |  | end | 16 | 0.01 (0.08) | -0.10 (0.21) | *.043** |
|  | Slow | Stroke | pre | post | 16 | 0.00 (0.05) | 0.06 (0.05) | *.005** |
|  |  |  |  | mid | 16 | 0.00 (0.06) | 0.08 (0.13) | *.087* |
|  |  |  |  | end | 16 | 0.01 (0.04) | 0.08 (0.15) | *.088* |
|  |  | Healthy | pre | post | 16 | -0.01 (0.03) | 0.07 (0.06) | *<.001** |
|  |  |  |  | mid | 16 | 0.01 (0.04) | 0.15 (0.09) | *<.001** |
|  |  |  |  | end | 16 | 0.01 (0.03) | 0.18 (0.11) | *<.001** |
| Cadence (stride/min) | Matched | Stroke | pre | post | 16 | -0.04 (1.22) | 0.21 (1.25) | *.162* |
|  |  |  |  | mid | 16 | 0.33 (2.21) | 0.56 (1.93) | *.050* |
|  |  |  |  | end | 15 | 0.53 (2.02) | -1.26 (2.25) | *.068* |
|  |  | Healthy | pre | post | 16 | 0.18 (0.53) | 0.06 (0.54) | *.455* |
|  |  |  |  | mid | 16 | 0.28 (1.03) | 1.32 (2.51) | *.083* |
|  |  |  |  | end | 15 | 0.75 (1.24) | 1.11 (1.65) | *.361* |
|  | Fast | Stroke | pre | post | 16 | -1.02 (1.59) | -2.41 (1.79) | *.841* |
|  |  |  |  | mid | 16 | -0.88 (2.55) | -2.12 (3.32) | *.051* |
|  |  |  |  | end | 15 | -1.07 (2.61) | -2.66 (3.74) | *.001** |
|  |  | Healthy | pre | post | 16 | -0.38 (0.44) | -1.41 (1.85) | *.030** |
|  |  |  |  | mid | 15 | -0.03 (0.95) | -0.41 (1.85) | *.302* |
|  |  |  |  | end | 14 | -0.04 (1.08) | -0.76 (2.29) | *.143* |
|  | Slow | Stroke | pre | post | 16 | 1.18 (1.27) | 1.44 (1.40) | *.377* |
|  |  |  |  | mid | 15 | 1.10 (2.81) | 1.13 (3.00) | *.393* |
|  |  |  |  | end | 13 | 1.23 (2.58) | 0.66 (4.50) | *.077* |
|  |  | Healthy | pre | post | 15 | 0.65 (0.63) | 1.16 (0.83) | *.068* |
|  |  |  |  | mid | 15 | 0.88 (0.93) | 2.35 (1.80) | *.008** |
|  |  |  |  | end | 14 | 1.13 (1.61) | 2.93 (2.26) | *.017** |
| Stride time (s) | Matched | Stroke | pre | post | 16 | 0.00 (0.04) | -0.01 (0.04) | *.378* |
|  |  |  |  | mid | 16 | -0.01 (0.06) | -0.02 (0.07) | *.562* |
|  |  |  |  | end | 15 | -0.03 (0.07) | 0.05 (0.08) | *.003** |
|  |  | Healthy | pre | post | 16 | 0.00 (0.01) | 0.00 (0.01) | *.491* |
|  |  |  |  | mid | 16 | -0.01 (0.2) | -0.03 (0.05) | *.081* |
|  |  |  |  | end | 15 | -0.02 (0.02) | -0.02 (0.03) | *.335* |
|  | Fast | Stroke | pre | post | 16 | 0.03 (0.05) | 0.10 (0.11) | *.030** |
|  |  |  |  | mid | 16 | 0.03 (0.09) | 0.08 (0.12) | *.083* |
|  |  |  |  | end | 15 | 0.03 (0.10) | 0.14 (0.32) | *.127* |
|  |  | Healthy | pre | post | 16 | 0.01 (0.01) | 0.04 (0.05) | *.050* |
|  |  |  |  | mid | 15 | 0.00 (0.02) | 0.01 (0.04) | *.219* |
|  |  |  |  | end | 14 | 0.00 (0.02) | 0.02 (0.05) | *.107* |
|  | Slow | Stroke | pre | post | 16 | -0.04 (0.12) | -0.05 (0.06) | *.581* |
|  |  |  |  | mid | 15 | -0.04 (0.12) | -0.01 (0.15) | *.642* |
|  |  |  |  | end | 13 | -0.05 (0.11) | -0.02 (0.16) | *.446* |
|  |  | Healthy | pre | post | 15 | -0.01 (0.01) | -0.02 (0.02) | *.030** |
|  |  |  |  | mid | 15 | -0.02 (0.02) | -0.05 (0.04) | *.009** |
|  |  |  |  | end | 14 | -0.02 (0.03) | -0.06 (0.05) | *.020** |

Values are reported in MD (mean difference) and SD (standard deviation). GRAIL: Gait Real-time Interactive Lab, HMD: head-mounted display. The asterisk indicates a significant difference.

**Additional table 3 continued.**

| Gait parameter | Condition | Time point | | N | GRAIL session  MD (SD) | HMD session  MD (SD) | GRAIL vs. HMD  p-value |
| --- | --- | --- | --- | --- | --- | --- | --- |
| Step length (cm) |  |  |  |  |  |  |  |
| *Affected leg post-stroke* | Matched | pre | post | 16 | 0.23 (2.03) | 1.33 (1.90) | *.193* |
|  |  |  | mid | 16 | 0.53 (2.96) | 2.02 (3.55) | *.155* |
|  |  |  | end | 15 | 1.97 (3.69) | 0.37 (3.85) | *.072* |
|  | Fast | pre | post | 16 | 0.43 (3.55) | -4.58 (4.30) | *<.001** |
|  |  |  | mid | 16 | 1.20 (5.17) | -2.13 (4.46) | *.030** |
|  |  |  | end | 15 | 2.36 (5.54) | -2.60 (6.55) | *.006** |
|  | Slow | pre | post | 16 | 2.09 (1.71) | 2.27 (2.03) | *.813* |
|  |  |  | mid | 15 | 2.68 (5.21) | 1.93 (4.71) | *.729* |
|  |  |  | end | 13 | 2.51 (3.77) | 2.89 (5.74) | *.827* |
| *Unaffected leg post-stroke* | Matched | pre | post | 16 | 0.10 (2.04) | 1.61 (2.72) | *.160* |
|  |  |  | mid | 16 | 0.83 (3.58) | 2.34 (4.21) | *.283* |
|  |  |  | end | 15 | 2.52 (5.37) | 0.66 (5.53) | *.164* |
|  | Fast | pre | post | 16 | 0.42 (3.84) | -5.01 (5.02) | *.001** |
|  |  |  | mid | 16 | -0.02 (4.99) | -2.50 (4.84) | *.099* |
|  |  |  | end | 15 | 0.57 (7.47) | -2.71 (8.01) | *.141* |
|  | Slow | pre | post | 16 | 1.79 (3.57) | 2.40 (2.94) | *.646* |
|  |  |  | mid | 15 | 3.10 (9.15) | 1.95 (6.43) | *.726* |
|  |  |  | end | 13 | 3.23 (8.39) | 3.12 (5.21) | *.967* |
| *Healthy (mean left-right)* | Matched | pre | post | 16 | 0.04 (1.63) | 1.16 (2.85) | *.265* |
|  |  |  | mid | 16 | 1.20 (2.66) | 2.67 (6.26) | *.452* |
|  |  |  | end | 15 | 1.39 (2.20) | 2.17 (4.15) | *.569* |
|  | Fast | pre | post | 16 | -1.55 (1.35) | -4.37 (3.63) | *.011** |
|  |  |  | mid | 15 | 0.36 (2.98) | -0.16 (2.75) | *.552* |
|  |  |  | end | 14 | 0.69 (2.91) | -1.74 (4.54) | *.039** |
|  | Slow | pre | post | 15 | 1.40 (1.52) | 1.74 (2.35) | *.616* |
|  |  |  | mid | 15 | 1.84 (2.12) | 4.09 (2.40) | *.010** |
|  |  |  | end | 14 | 1.57 (1.77) | 5.14 (2.72) | *<.001** |
| Step width (cm) |  |  |  |  |  |  |  |
| *Affected leg post-stroke* | Matched | pre | post | 16 | 0.01 (1.62) | 0.03 (0.91) | *.967* |
|  |  |  | mid | 16 | 0.22 (0.89) | 0.33 (0.92) | *.624* |
|  |  |  | end | 15 | 0.76 (1.71) | 0.39 (1.07) | *.417* |
|  | Fast | pre | post | 16 | 0.14 (0.80) | -0.59 (1.15) | *.049** |
|  |  |  | mid | 16 | 0.09 (0.97) | -0.29 (1.14) | *.299* |
|  |  |  | end | 15 | 0.74 (1.35) | 0.07 (1.55) | *.208* |
|  | Slow | pre | post | 16 | 0.35 (0.72) | 0.44 (1.18) | *.813* |
|  |  |  | mid | 15 | 0.62 (1.89) | 0.26 (1.31) | *.591* |
|  |  |  | end | 13 | 0.64 (1.75) | 0.67 (0.79) | *.946* |
| *Unaffected leg post-stroke* | Matched | pre | post | 16 | -0.02 (1.44) | 0.00 (0.89) | *.956* |
|  |  |  | mid | 16 | 0.27 (0.84) | 0.34 (0.85) | *.710* |
|  |  |  | end | 15 | 0.78 (1.66) | 0.32 (1.16) | *.334* |
|  | Fast | pre | post | 16 | 0.01 (0.82) | -0.56 (1.33) | *.201* |
|  |  |  | mid | 16 | 0.09 (0.99) | -0.18 (1.22) | *.427* |
|  |  |  | end | 15 | 0.69 (1.33) | 0.07 (1.48) | *.227* |
|  | Slow | pre | post | 16 | 0.34 (0.69) | 0.40 (1.15) | *.849* |
|  |  |  | mid | 15 | 0.49 (1.87) | 0.18 (1.24) | *.630* |
|  |  |  | end | 13 | 0.54 (1.64) | 0.73 (0.80) | *.720* |
| *Healthy (mean left-right)* | Matched | pre | post | 16 | 0.18 (0.93) | 0.06 (0.86) | *.702* |
|  |  |  | mid | 16 | 0.06 (0.89) | 0.15 (0.86) | *.759* |
|  |  |  | end | 15 | 0.07 (0.87) | 0.27 (1.01) | *.583* |
|  | Fast | pre | Post | 16 | 0.49 (0.54) | 0.46 (0.86) | *.906* |
|  |  |  | mid | 15 | 0.26 (1.20) | 0.40 (0.72) | *.580* |
|  |  |  | end | 14 | 0.07 (1.69) | 0.60 (1.09) | *.237* |
|  | Slow | pre | post | 15 | 0.07 (1.07) | 0.09 (0.88) | *.953* |
|  |  |  | mid | 15 | -0.06 (0.95) | 0.25 (1.20) | *.331* |
|  |  |  | end | 14 | 0.12 (0.82) | 0.43 (0.91) | *.303* |

Values are reported in MD (mean difference) and SD (standard deviation). GRAIL: Gait Real-time Interactive Lab, HMD: head-mounted display. The asterisk indicates a significant difference.

**Additional Table 3 continued.**

| Gait parameter | Condition | Time point | | N | GRAIL session  MD (SD) | HMD session  MD (SD) | GRAIL vs. HMD  p-value |
| --- | --- | --- | --- | --- | --- | --- | --- |
| Stance time (%GC) |  |  |  |  |  |  |  |
| *Affected leg post-stroke* | Matched | pre | post | 16 | 0.08 (0.69) | -0.27 (0.68) | *.147* |
|  |  |  | mid | 16 | -0.25 (1.06) | -0.49 (1.02) | *.341* |
|  |  |  | end | 15 | -0.65 (1.66) | 0.06 (1.47) | *0.022** |
|  | Fast | pre | post | 16 | -0.19 (1.29) | 0.37 (2.37) | *.251* |
|  |  |  | mid | 16 | 0.39 (0.99) | 0.87 (1.45) | *.166* |
|  |  |  | end | 15 | -0.03 (1.27) | 1.04 (2.27) | *.075* |
|  | Slow | pre | post | 16 | -0.88 (1.09) | -0.35 (1.07) | *.258* |
|  |  |  | mid | 15 | -1.28 (2.54) | -0.69 (2.32) | *.580* |
|  |  |  | end | 13 | -1.08 (2.27) | -0.49 (2.15) | *.482* |
| *Unaffected leg post-stroke* | Matched | pre | post | 16 | -0.09 (0.71) | -0.19 (0.60) | *.625* |
|  |  |  | mid | 16 | -0.08 (0.92) | -0.40 (1.21) | *.258* |
|  |  |  | end | 15 | -0.56 (1.31) | 0.18 (1.12) | *.023** |
|  | Fast | pre | post | 16 | 0.28 (0.94) | 1.64 (1.70) | *.025** |
|  |  |  | mid | 16 | 0.37 (1.35) | 0.83 (0.97) | *.181* |
|  |  |  | end | 15 | 0.19 (1.55) | 1.15 (1.72) | *.020** |
|  | Slow | pre | post | 16 | -0.53 (0.83) | -0.84 (0.85) | *.162* |
|  |  |  | mid | 15 | -0.44 (1.48) | -0.55 (1.62) | *.843* |
|  |  |  | end | 13 | -0.49 (1.00) | -0.77 (1.68) | *.483* |
| *Healthy (mean left-right)* | Matched | pre | post | 16 | 0.04 (0.28) | -0.15 (0.29) | *.118* |
|  |  |  | mid | 16 | -0.08 (0.46) | -0.34 (0.77) | *.334* |
|  |  |  | end | 15 | -0.19 (0.41) | -0.27 (0.43) | *.648* |
|  | Fast | pre | post | 16 | 0.30 (0.34) | 0.82 (0.74) | *.033** |
|  |  |  | mid | 15 | 0.00 (0.49) | 0.15 (0.57) | *.314* |
|  |  |  | end | 14 | 0.04 (0.53) | 0.43 (0.85) | *.083* |
|  | Slow | pre | post | 15 | -0.27 (0.35) | -0.33 (0.45) | *.540* |
|  |  |  | mid | 15 | -0.30 (0.35) | -0.71 (0.47) | *.008** |
|  |  |  | end | 14 | -0.32 (0.41) | -0.93 (0.52) | *.002** |
| Swing time (%GC) |  |  |  |  |  |  |  |
| *Affected leg post-stroke* | Matched | pre | post | 16 | -0.09 (0.67) | 0.28 (0.66) | *.107* |
|  |  |  | mid | 16 | 0.18 (1.06) | 0.43 (1.02) | *.288* |
|  |  |  | end | 15 | 0.63 (1.60) | -0.11 (1.42) | *.012** |
|  | Fast | pre | post | 16 | 0.20 (1.24) | -0.53 (2.37) | *.142* |
|  |  |  | mid | 16 | -0.29 (1.04) | -0.83 (1.41) | *.104* |
|  |  |  | end | 15 | 0.16 (1.73) | -0.98 (2.20) | *.044** |
|  | Slow | pre | post | 16 | 0.81 (1.09) | 0.36 (1.04) | *.307* |
|  |  |  | mid | 15 | 1.18 (2.48) | 0.70 (2.24) | *.635* |
|  |  |  | end | 13 | 1.03 (2.18) | 0.42 (2.08) | *.428* |
| *Unaffected leg post-stroke* | Matched | pre | post | 16 | 0.09 (0.71) | 0.19 (0.60) | *.625* |
|  |  |  | mid | 16 | 0.08 (0.92) | 0.40 (1.21) | *.258* |
|  |  |  | end | 15 | 0.56 (1.31) | -0.18 (1.12) | *.023** |
|  | Fast | pre | post | 16 | -0.28 (0.94) | -1.46 (1.70) | *.025** |
|  |  |  | mid | 16 | -0.37 (1.35) | -0.83 (0.97) | *.181* |
|  |  |  | end | 15 | -0.19 (1.55) | -1.15 (1.72) | *.020** |
|  | Slow | pre | post | 16 | 0.53 (0.83) | 0.84 (0.85) | *.162* |
|  |  |  | mid | 15 | 0.44 (1.48) | 0.55 (11.62) | *.843* |
|  |  |  | end | 13 | 0.49 (1.00) | 0.77 (1.68) | *.483* |
| *Healthy (mean left-right)* | Matched | pre | post | 16 | -0.04 (0.28) | 0.15 (0.29) | *.118* |
|  |  |  | mid | 16 | 0.08 (0.46) | 0.34 (0.77) | *.334* |
|  |  |  | end | 15 | 0.19 (0.41) | 0.27 (0.43) | *.648* |
|  | Fast | pre | post | 16 | -0.30 (0.34) | -0.82 (0.74) | *.033** |
|  |  |  | mid | 15 | 0.00 (0.49) | -0.15 (0.57) | *.314* |
|  |  |  | end | 14 | -0.04 (0.53) | -0.43 (0.85) | *.083* |
|  | Slow | pre | post | 15 | 0.27 (0.35) | 0.33 (0.45) | *.540* |
|  |  |  | mid | 15 | 0.30 (0.35) | 0.71 (0.47) | *.008** |
|  |  |  | end | 14 | 0.32 (0.41) | 0.93 (0.52) | *.002** |

Values are reported in MD (mean difference) and SD (standard deviation). GRAIL: Gait Real-time Interactive Lab, HMD: head-mounted display, %GC: percentage gait cycle. The asterisk indicates a significant difference.

**Additional table 4: Linear mixed models for the Igroup Presence Questionnaire**

| Spatiotemporal gait parameter | LMM | Fixed factor(s) | AIC |
| --- | --- | --- | --- |
| General item | 1 | **Condition** | 231.47 |
|  | 2 | Condition * Group | 234.92 |
| Spatial presence | 1 | Condition | 132.43 |
|  | 2 | **Condition * Group** | 130.24 |
| Involvement | 1 | **Condition** | 220.06 |
|  | 2 | Condition * Group | 223.15 |
| Experienced realism | 1 | **Condition** | 190.99 |
|  | 2 | Condition * Group | 193.73 |

LMM: Linear Mixed-Effects Models. AIC: Akaike’s Information Criterion (smaller values indicate a better model).

**Additional figure 1: 2-way ANOVA SPM analyses for the joint angles (affected side post-stroke)**


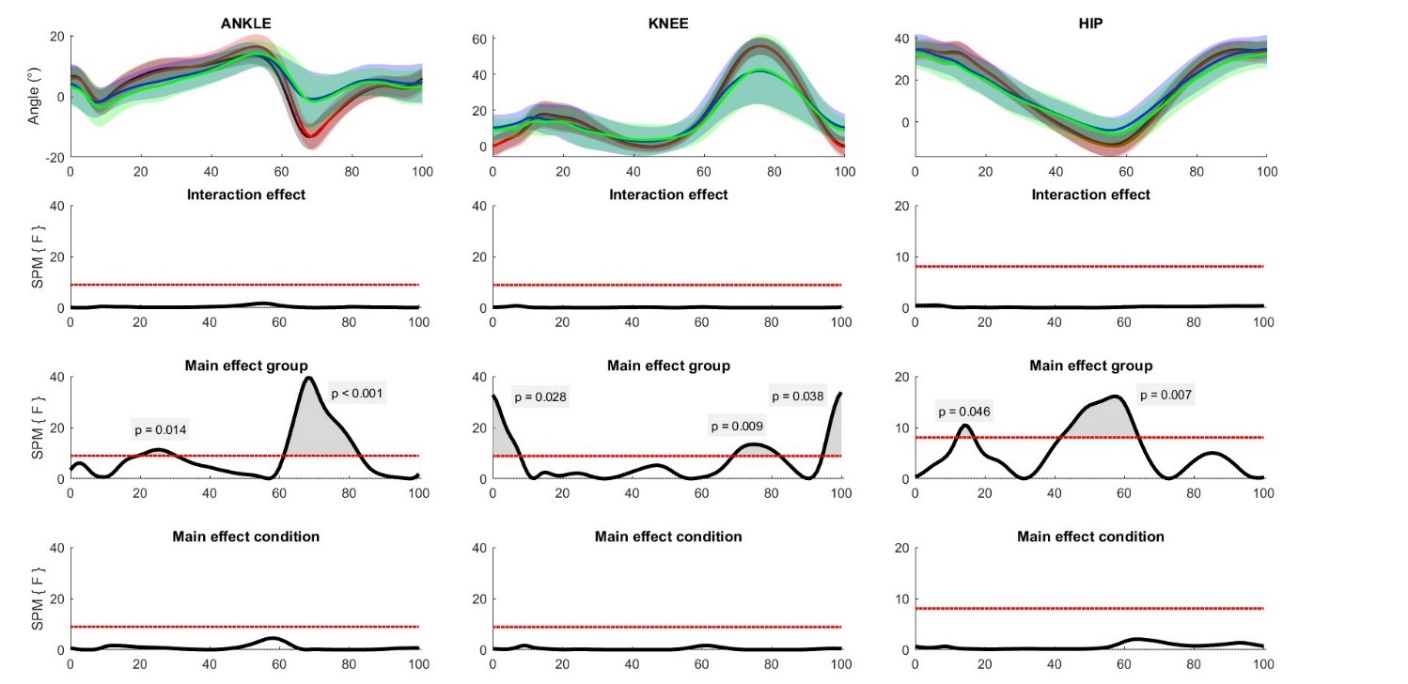


Horizontal axis is percentage gait cycle. First row is mean joint angles ± 1 standard deviation for healthy people with no VR (black), healthy people with VR (red), people post-stroke (affected side) with no VR (blue), people post-stroke (affected side) with VR (green). Second to fourth rows show SPM(F) value throughout the gait cycle. Dashed red lines is equivalent to α=0.05.

**Additional figure 2: 2-way ANOVA SPM analyses for the joint angles (unaffected side post-stroke)**


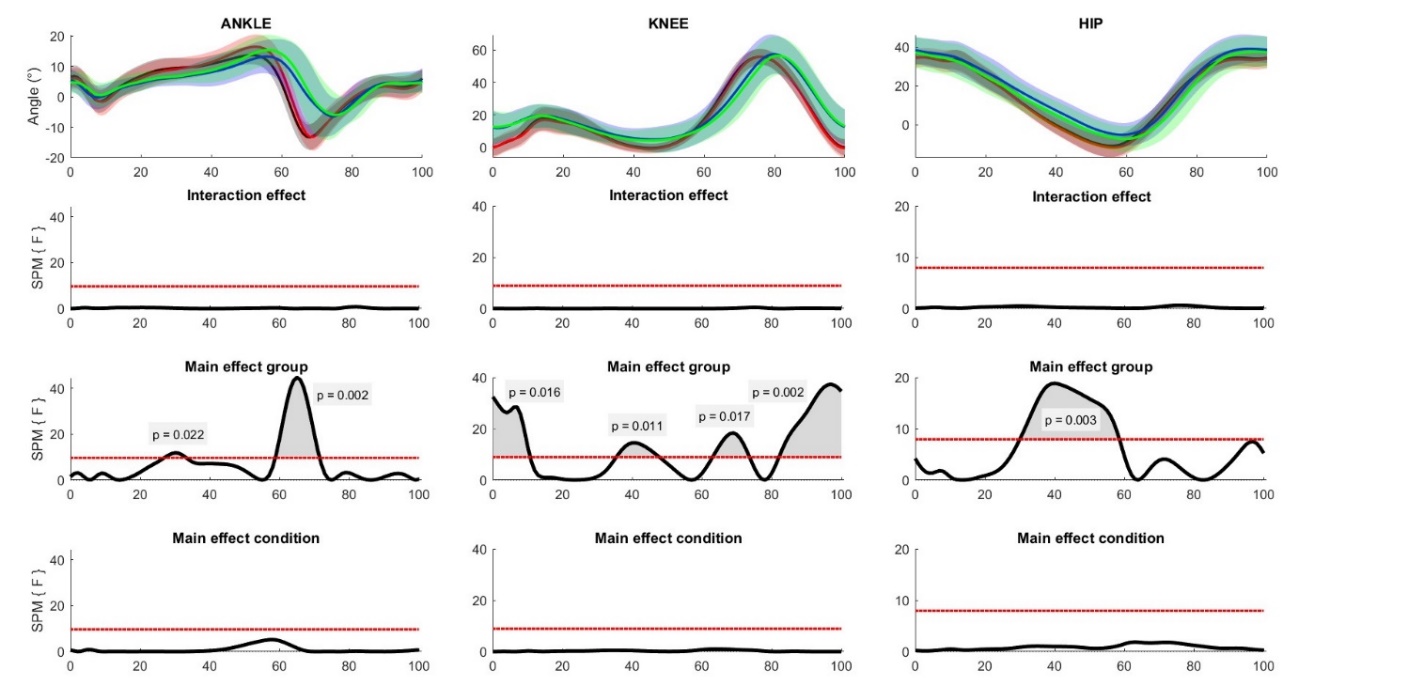


Horizontal axis is percentage gait cycle. First row is mean joint angles ± 1 standard deviation for healthy people with no VR (black), healthy people with VR (red), people post-stroke (unaffected side) with no VR (blue), people post-stroke (unaffected side) with VR (green). Second to fourth rows show SPM(F) value throughout the gait cycle. Dashed red lines is equivalent to α=0.05.

**Additional figure 3: 2-way repeated measures ANOVA SPM analyses matched condition (affected side post stroke)**


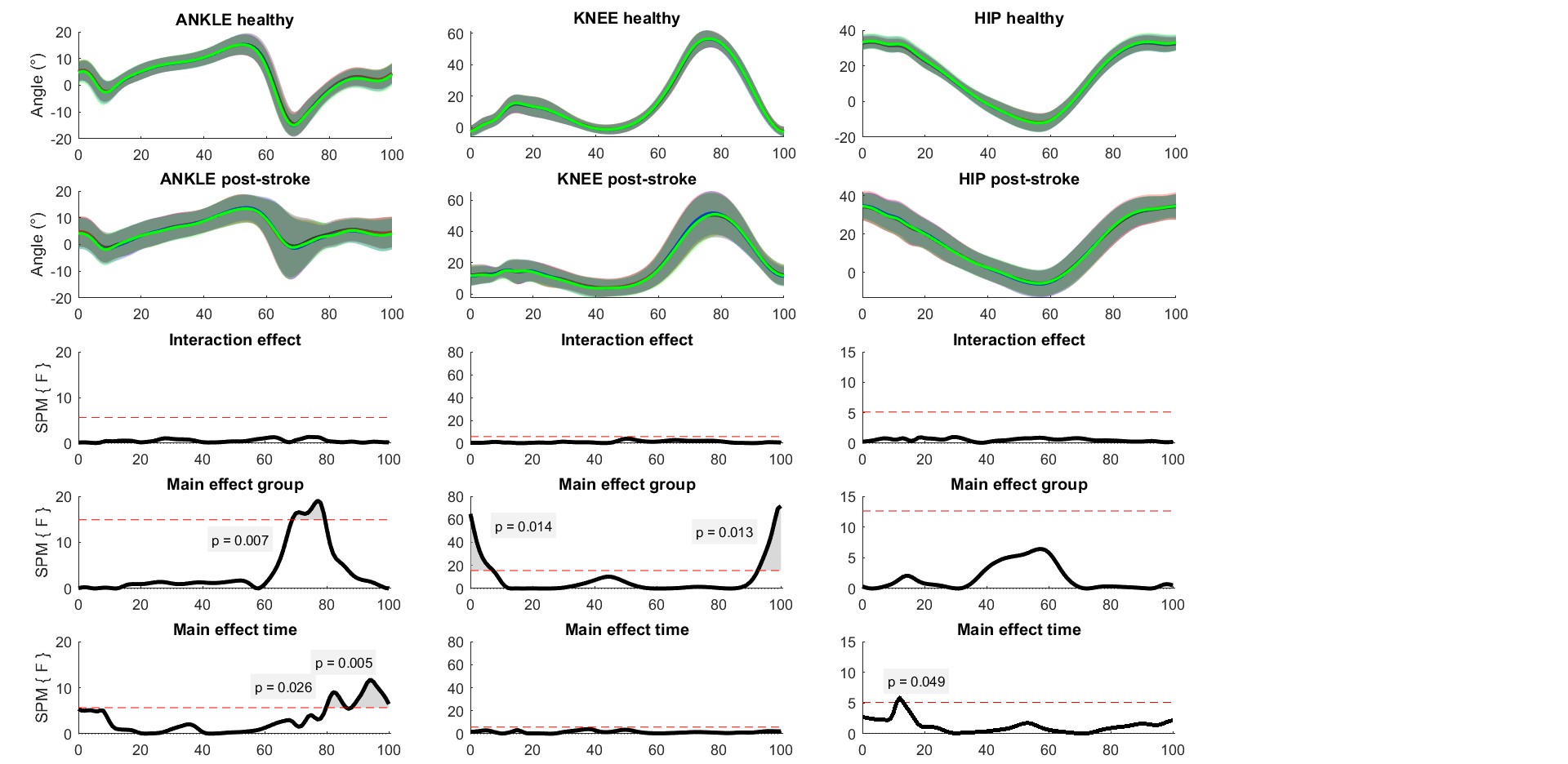


Horizontal axis is percentage gait cycle. First two rows are mean joint angles ± 1 standard deviation for healthy people and people post-stroke pre manipulation (black), post manipulation (red), middle 30 sec (blue) and last 30 sec (green). Third to fifth rows show SPM(F) value throughout the gait cycle. Dashed red lines is equivalent to α=0.05.

**Additional figure 4: 2-way repeated measures ANOVA SPM analyses fast condition (affected side post stroke)**


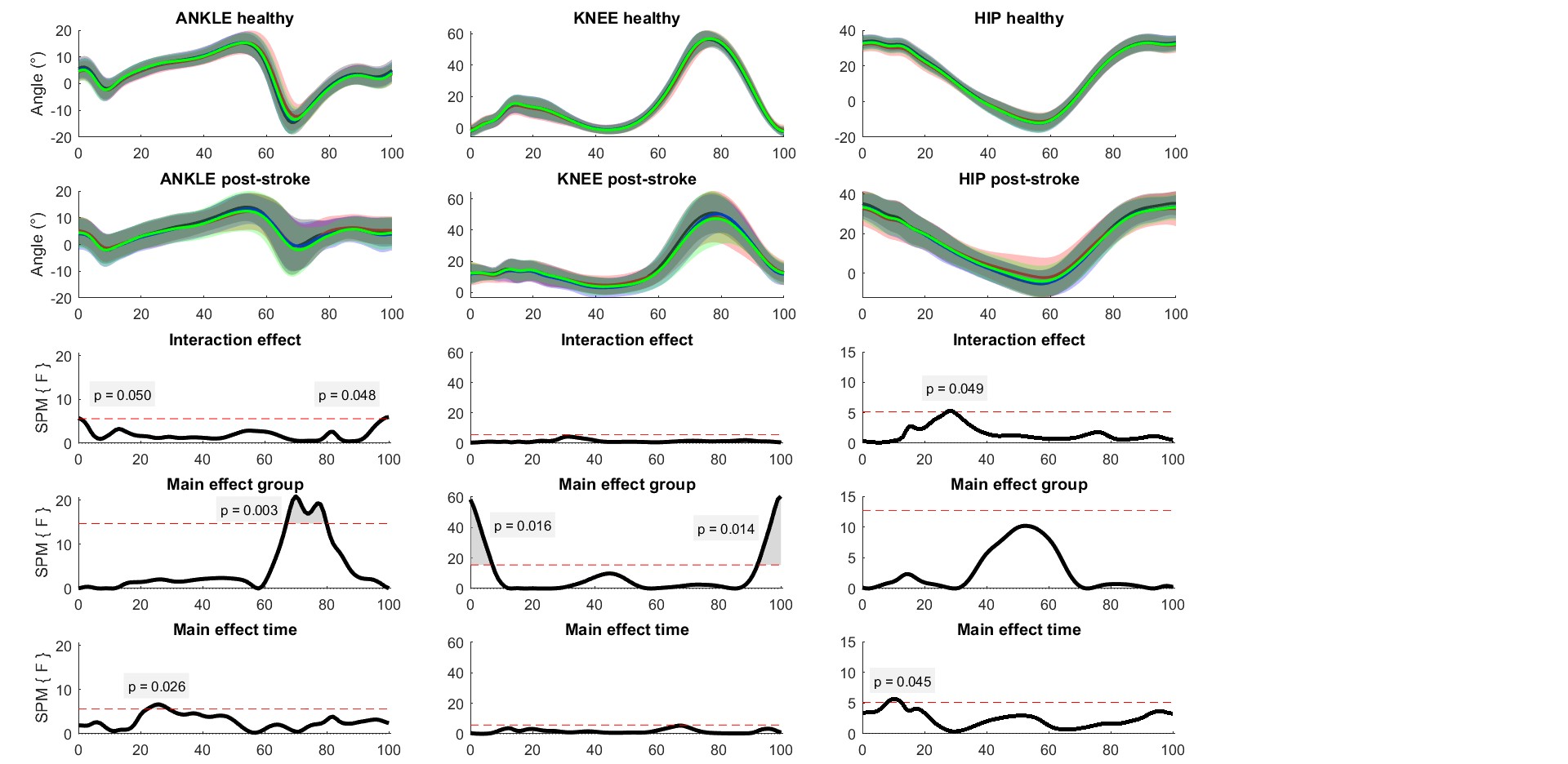


Horizontal axis is percentage gait cycle. First two rows are mean joint angles ± 1 standard deviation for healthy people and people post-stroke pre manipulation (black), post manipulation (red), middle 30 sec (blue) and last 30 sec (green). Third to fifth rows show SPM(F) value throughout the gait cycle. Dashed red lines is equivalent to α=0.05.

**Additional figure 5: 2-way repeated measures ANOVA SPM analyses slow condition (affected side post stroke)**


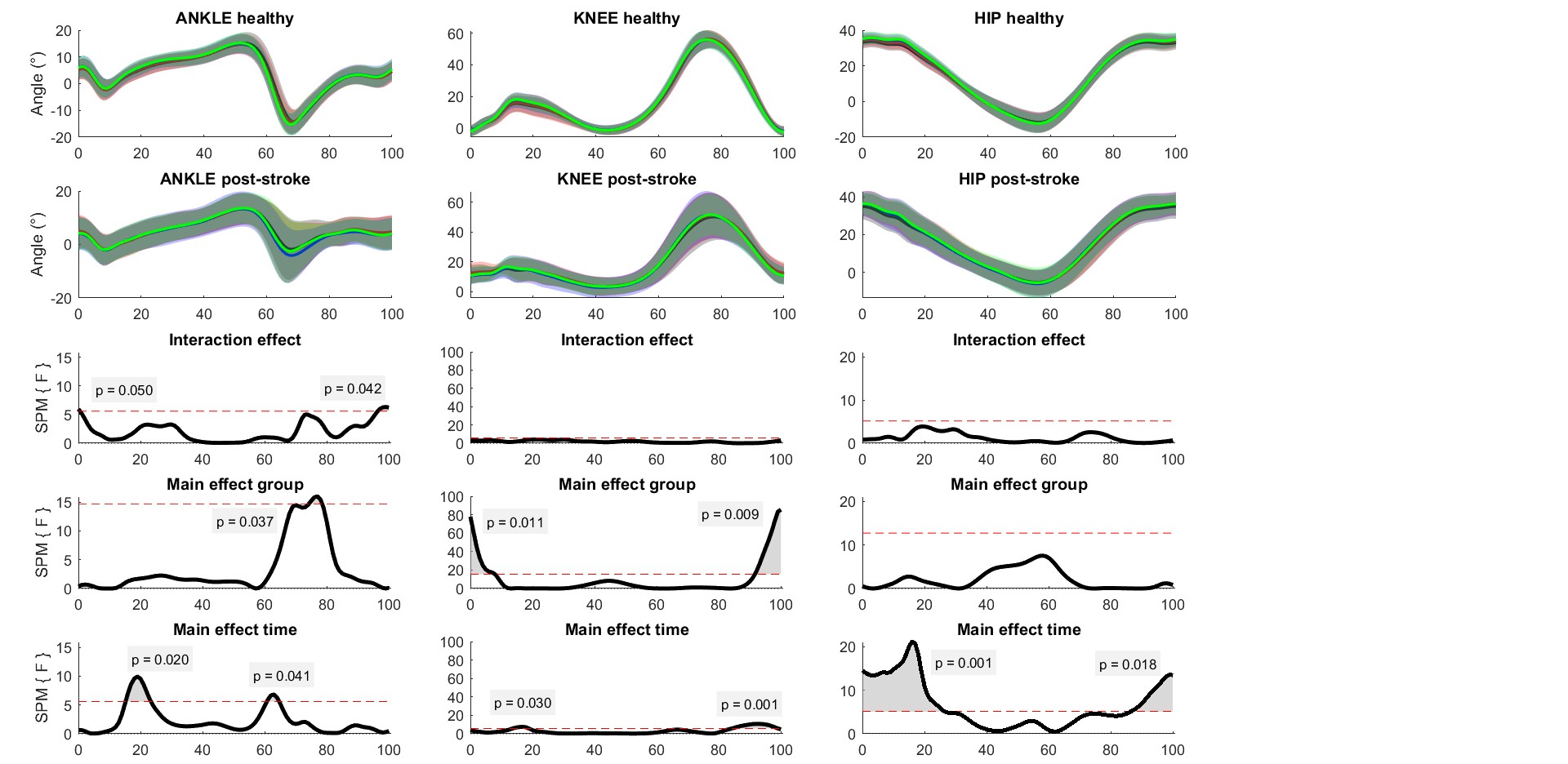


Horizontal axis is percentage gait cycle. First two rows are mean joint angles ± 1 standard deviation for healthy people and people post-stroke pre manipulation (black), post manipulation (red), middle 30 sec (blue) and last 30 sec (green). Third to fifth rows show SPM(F) value throughout the gait cycle. Dashed red lines is equivalent to α=0.05.

**Additional figure 6: 2-way repeated measures ANOVA SPM analyses matched condition (unaffected side post stroke)**

**
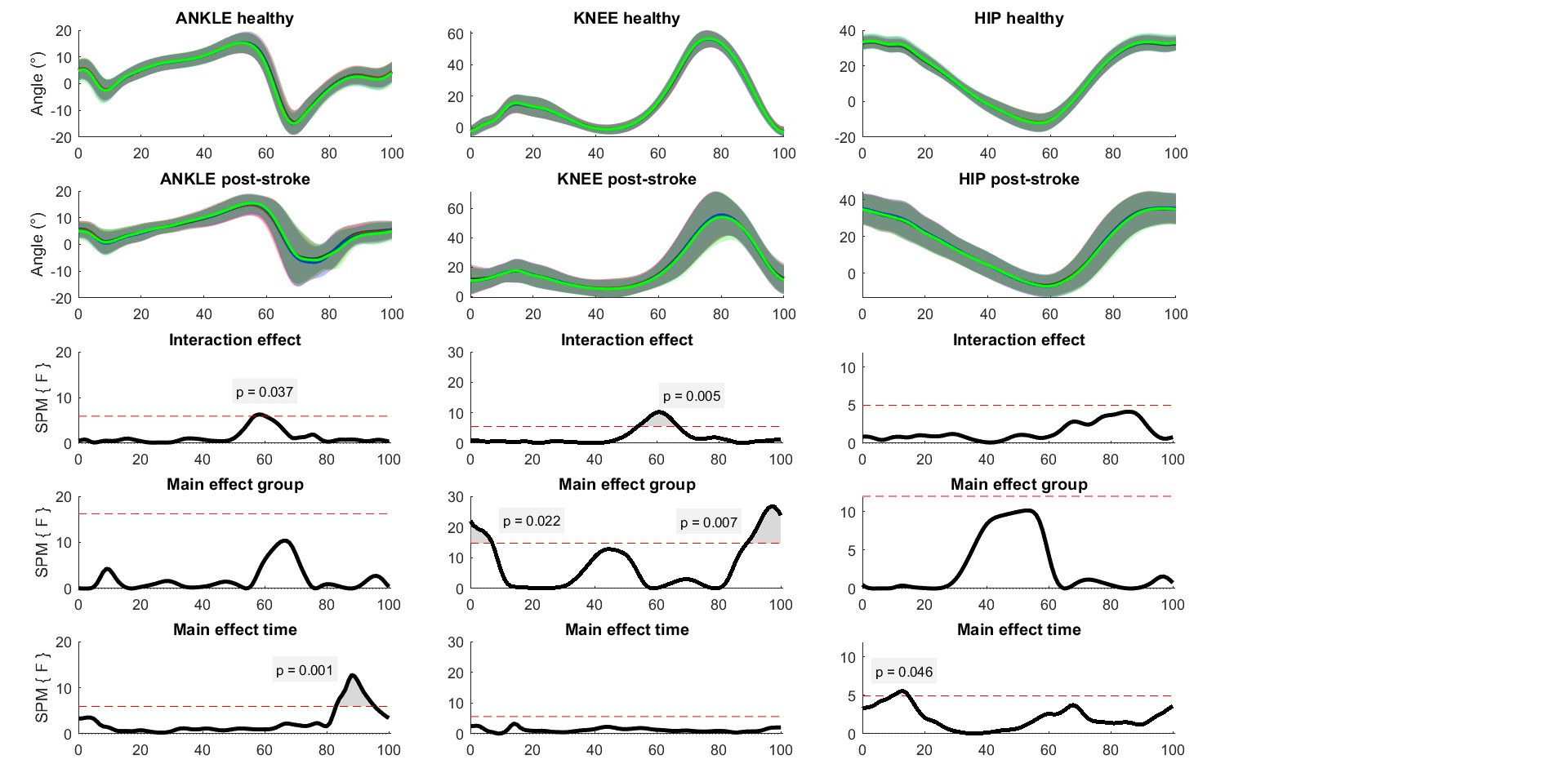
**

Horizontal axis is percentage gait cycle. First two rows are mean joint angles ± 1 standard deviation for healthy people and people post-stroke pre manipulation (black), post manipulation (red), middle 30 sec (blue) and last 30 sec (green). Third to fifth rows show SPM(F) value throughout the gait cycle. Dashed red lines is equivalent to α=0.05.

**Additional figure 7: 2-way repeated measures ANOVA SPM analyses fast condition (unaffected side post stroke)**

**
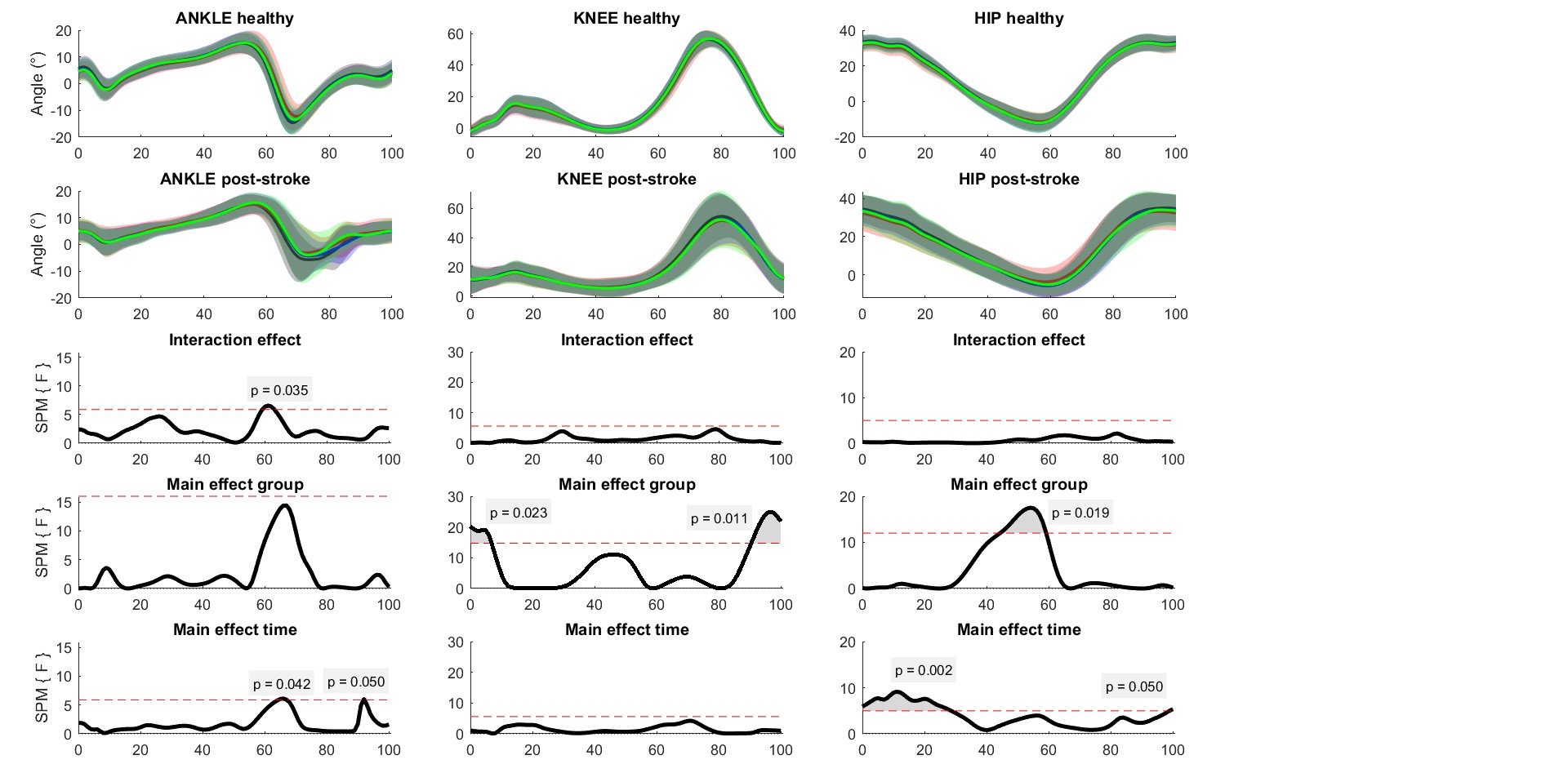
**

Horizontal axis is percentage gait cycle. First two rows are mean joint angles ± 1 standard deviation for healthy people and people post-stroke pre manipulation (black), post manipulation (red), middle 30 sec (blue) and last 30 sec (green). Third to fifth rows show SPM(F) value throughout the gait cycle. Dashed red lines is equivalent to α=0.05.

**Additional figure 8: 2-way repeated measures ANOVA SPM analyses slow condition (unaffected side post stroke)**

**
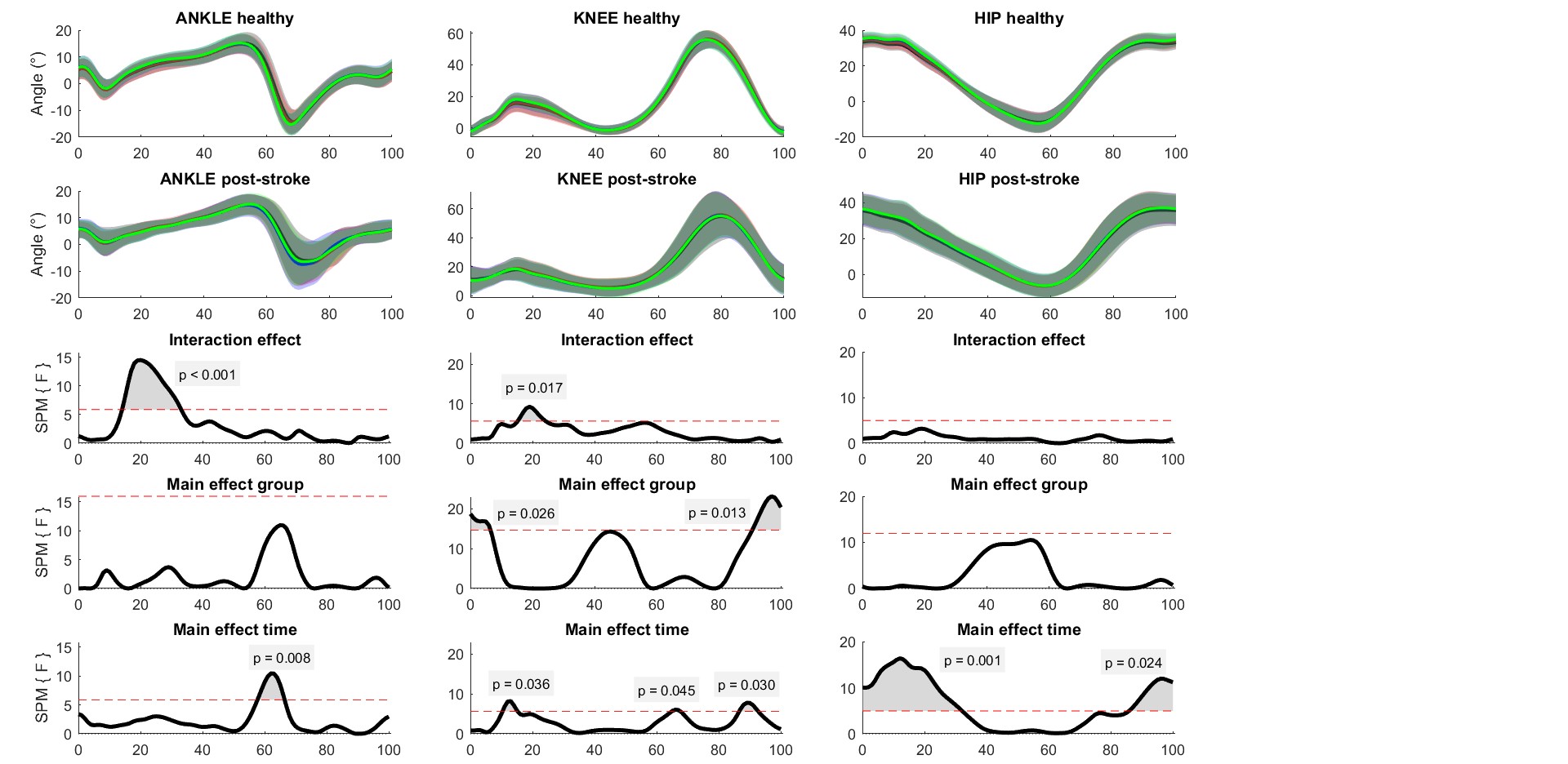
**

Horizontal axis is percentage gait cycle. First two rows are mean joint angles ± 1 standard deviation for healthy people and people post-stroke pre manipulation (black), post manipulation (red), middle 30 sec (blue) and last 30 sec (green). Third to fifth rows show SPM(F) value throughout the gait cycle. Dashed red lines is equivalent to α=0.05.
